# Short-term effects of air pollution on a range of cardiovascular events in England and Wales: case-crossover analysis of the MINAP database, hospital admissions and mortality

**DOI:** 10.1136/heartjnl-2013-304963

**Published:** 2014-06-04

**Authors:** Ai Milojevic, Paul Wilkinson, Ben Armstrong, Krishnan Bhaskaran, Liam Smeeth, Shakoor Hajat

**Affiliations:** 1 Department of Social and Environmental Health Research, London School of Hygiene and Tropical Medicine, London, UK; 2 Department of Non-Communicable Disease Epidemiology, London School of Hygiene and Tropical Medicine, London, UK

**Keywords:** MYOCARDIAL ISCHAEMIA AND INFARCTION (IHD), ARRHYTHMIAS

## Abstract

**Objective:**

To inform potential pathophysiological mechanisms of air pollution effects on cardiovascular disease (CVD), we investigated short-term associations between ambient air pollution and a range of cardiovascular events from three national databases in England and Wales.

**Methods:**

Using a time-stratified case-crossover design, over 400 000 myocardial infarction (MI) events from the Myocardial Ischaemia National Audit Project (MINAP) database, over 2 million CVD emergency hospital admissions and over 600 000 CVD deaths were linked with daily mean concentrations of carbon monoxide (CO), nitrogen dioxide (NO_2_), particulate matter less than 10 μm in aerodynamic diameter (PM_10_), particulate matter less than 2.5 μm in aerodynamic diameter (PM_2.5_) and sulfur dioxide (SO_2_), and daily maximum of 8-hourly running mean of O_3_ measured at the nearest air pollution monitoring site to the place of residence. Pollutant effects were modelled using lags up to 4 days and adjusted for ambient temperature and day of week.

**Results:**

For mortality, no CVD outcome analysed was clearly associated with any pollutant, except for PM_2.5_ with arrhythmias, atrial fibrillation and pulmonary embolism. With hospital admissions, only NO_2_ was associated with a raised risk: CVD 1.7% (95% CI 0.9 to 2.6), non-MI CVD 2.0% (1.1 to 2.9), arrhythmias 2.9% (0.6 to 5.2), atrial fibrillation 2.8% (0.3 to 5.4) and heart failure 4.4% (2.0 to 6.8) for a 10th–90th centile increase. With MINAP, only NO_2_ was associated with an increased risk of MI, which was specific to non-ST-elevation myocardial infarction (non-STEMIs): 3.6% (95% CI 0.4 to 6.9).

**Conclusions:**

This study found no clear evidence for pollution effects on STEMIs and stroke, which ultimately represent thrombogenic processes, though it did for pulmonary embolism. The strongest associations with air pollution were observed with selected non-MI outcomes.

## Introduction

Experimental and epidemiological studies have provided evidence of associations between air pollution and cardiovascular health.^1–3^ More pollution-related deaths occur from heart disease than from any other cause. A comparative risk assessment concluded that 7.4% of all cases of myocardial infarction (MI) is attributable to traffic-related air pollution,^4^ and a recent systematic literature review found associations with MI to be significant with all pollutants except O_3._^5^

Uncertainties remain, however, about the likely mechanisms of pollution-related cardiovascular disease (CVD). For example, hypotheses for particulate pollution include disturbance of the autonomic nervous system,^6^ changes in blood coagulability consequent to alveolar inflammation^7^ and the translocation of particles and/or their constituents into the blood.^3^

This study aims to further current understanding of pathophysiological mechanisms by examining the strength and specificity of acute relationships between ambient air pollution and a range of CVD events. The key mechanistic question addressed is whether events of clear thrombotic origin, namely, acute MI, stroke and related outcomes, have a stronger association with air pollution than non-thrombotic outcomes.

## Methods

### Health data

We analysed data from three databases: the Myocardial Ischaemia National Audit Project (MINAP) database, Hospital Episode Statistics (HES) and mortality (Office for National Statistics).

MINAP is a national register of admissions to hospital of patients with acute coronary syndrome/MI. All 230 acute hospitals in England and Wales contribute to the database, giving it theoretically complete geographical coverage. The database includes information on patient characteristics (including age, sex, smoking status), the timing of onset of symptoms, diagnostic data (ECG changes, enzyme markers, symptoms, etc.), previously recognised CVD, comorbidity (hypertension, diabetes, asthma, COPD, etc.), acute treatment, current therapy (such as aspirin or statins) and fatal outcome. Analysis consisted of all events during 2003–2009 that contained location information rounded to 100 m resolution of the centroid of the patient's enumeration district of residence. On average, enumeration districts contain 450 residents or 200 households. All patients with a discharge diagnosis of ST elevation MI (STEMI), non-ST elevation MI (non-STEMI), or troponin-positive acute coronary syndrome were included. Diagnosis of STEMI at discharge was based on clinical history, presence of cardiographic changes of ST elevation consistent with infarction and elevated enzyme or troponin levels. Diagnosis of non-STEMI was based on symptoms consistent with cardiac ischaemia, other cardiographic changes and elevated troponin levels.

The HES database consists of routine statistics on all admissions made to NHS hospitals in England and Wales. We analysed data on emergency admissions during 2003–2008. The geographical marker used for patient's residence was the population-weighted centroid of census ward, which, on average, contains 6000 people. Mortality data included all nationwide CVD deaths during 2003–2006, with location based on the centroid of the postcode of residence. For both HES and mortality, the outcomes analysed were all CVD (ICD10 codes I00-I99), MI (I21-I23), all CVD except MI (referred to as non-MI CVD), stroke (I60-I69), ischaemic heart disease (IHD, I20-I25), chronic IHD (I25), pulmonary embolism (I26), atrioventricular conduction disorders (I44, I45), arrhythmias (I47, I48), atrial fibrillation (I48) and heart failure (I50).

### Exposure data

Daily 24-h average values of particulate matter (particulate matter less than 10 μm in aerodynamic diameter (PM_10_) and particulate matter less than 2.5 μm in aerodynamic diameter (PM_2.5_)), carbon monoxide (CO), nitrogen dioxide (NO_2_) and sulfur dioxide (SO_2_), and daily maximum values of 8-h running mean of ozone (O_3_), excluding roadside and kerbside sites, were obtained from monitoring stations run by the UK National Air Quality Information Archive (see online supplementary figure S1). Exposure was characterised for each health event using the nearest monitoring station to the place of residence, with the condition that exposure information was not missing on the event day and on ≥90% of days within each risk period (as defined in the analysis section). Based on preliminary analysis of season-specific correlations and mean differences between monitors at varying distances (see online supplementary figure S2), 50 km was set as the maximum distance within which to characterise exposure. In the rare situation where no station resided within 50 km, that event was excluded from analysis.

Weather data were obtained for all monitoring stations via the British Atmospheric Data Centre (UK Meteorological Office MIDAS Land Surface Stations Data). Daily maximum and minimum temperatures were averaged to derive daily mean temperature. Linkage of health events to weather stations was based on the same algorithm as for air pollution.

### Analysis

Short-term associations between air pollution and CVD events were assessed using a time-stratified case-crossover approach, whereby the day of each health event is considered as the case and all other days within the same month as controls. Lunar month was used for stratification to ensure periods of equal duration (ie, 28 days). Conditional logistic regression was used to assess associations, using pollutant measures lagged by up to 4 days (unconstrained distributed lag model). To maximise power, day-of-week effects were controlled for using indicator variables rather than the more common approach of matching.^8^ ^9^ However, for the two most numerous outcomes (all CVD and non-MI CVD admissions), this proved computationally limiting, and so matching was used instead.

The potentially confounding effects of temperature were controlled for using natural cubic splines (3 knots) of mean temperature, lagged by up to 2 weeks based on previous evidence.^10^ Potential autocorrelation in the event data was assessed and allowed for by introducing as explanatory variables residuals lagged by 1 and 2 days in all models. Effect modification of risk by age and sex was explored, and for the MINAP analysis, additional modifiers were also examined.

Several sensitivity analyses were performed. First, where a strong pollution effect was observed, a second pollutant was incorporated to assess independent effects of the primary pollutant. Second, seasonal effects were examined by restricting analysis to summer months (June–August) for O_3_. Third, we considered a shorter lag structure of 0–1 days. Fourth, we allowed for day-of-week effects by matching rather than explicit control. Fifth, main analyses were repeated using robust SEs to allow for possible clustering by monitoring sites. Analyses were conducted using STATA V.12. Ethics approval was granted by the Ethics Committee of LSHTM.

## Results

Summary statistics for the exposure and health data are presented in tables 1 and 2, respectively.

**Table 1:** Summary of exposure data in 2003–2009

Pollutant	Number of monitors	Median (IQR)	10th–90th centile range
CO (mg/m^3^)	61	0.2 (0.2–0.4)	0.4
NO_2_ (μg/m^3^)	93	24 (13–37)	45
O_3_ (μg/m^3^)	82	61 (46–76)	61
PM_10_ (μg/m^3^)	62	20 (15–27)	26
PM_2.5_ (μg/m^3^)	46	10 (7–15)	16
SO_2_ (μg/m^3^)	71	3.1 (2–6)	10.4
Mean temperature (d/C)	717	9.85 (6.2–13.9)	13.85

CO, carbon monoxide; NO_2_, nitrogen dioxide; PM_2.5_, particulate matter less than 2.5 μm in aerodynamic diameter; PM_10_, particulate matter less than 10 μm in aerodynamic diameter; SO_2_, sulfur dioxide.

**Table 2:** Summary statistics and number of events for the MINAP registries in 2003–2009, emergency hospital admissions from HES in 2003–2008 and ONS mortality in 2003–2006

	MINAP	HES	ONS mortality
Age (years): median (IQR)	71 (60–81)	73 (60–82)	82 (74–88)
Male (%)	65	54	48
All CVD events (N)		2 867 473	752 004
Stroke		461 845	209 294
IHD		967 677	361 738
MI	452 343	417 833	151 483
Chronic IHD		85 989	208 505
Arrhythmias		379 605	11 703
Atrial fibrillation		310 568	11 587
AVCD		47 666	463
Pulmonary embolism		88 988	12 520
Heart failure		335 495	37 033
Sudden (cardiac) death		532	90
Pulmonary heart disease		2000	603

AVCD, atrioventricular conduction disorders; CVD, cardiovascular disease; HES, Hospital Episode Statistics; IHD, ischaemic heart disease; MI, myocardial infarction; MINAP, Myocardial Ischaemia National Audit Project; ONS, Office for National Statistics.

### MINAP

There were 452 343 geographically coded MI events during the study period. Patients with non-STEMI events were older than STEMI patients (age 70+ years 60.4% vs 41.6%, respectively) and had higher rates of comorbidity (previous MI 30.1% vs 14.4%; previous angina 35.9% vs 16.3%; previous hypertension 47.4% vs 36.4%; and diabetes 20.8% vs 11.9%, see online supplementary table S1).

Table 3 presents the percent change in MI risk for 10th–90th centile increases in pollutant measures, for all MI events and separately for STEMI (approximately 42% of the cases) and non-STEMI diagnosis. In general, there was very little evidence of a pollution effect on MI risk, with as many negative effect estimates as positive. The strongest evidence for any adverse effect was with NO_2_, where a 10th–90th centile increase was associated with a 2.4% increase (95% CI 0.3 to 4.5%) in overall MI risk, and with SO_2_ where the corresponding increase was 1.7% (−0.1 to 3.4%). Associations for these two pollutants appeared to be stronger for non-STEMI compared with STEMI events. There were few factors that consistently modified the risk of NO_2_ or SO_2_ on either STEMIs or non-STEMIs (see online supplementary figures S3 and S4). The effects on non-STEMIs were significantly greater when the outcome was fatal compared with non-fatal. Those aged 70+ years were also at heightened risk, and those with a previous percutaneous coronary intervention were at reduced risk of MI following exposure to either pollutant.

**Table 3:** Number of events linked to each pollutant data and per cent change in risk* of MI for all MI, STEMI and non-STEMI diagnoses associated with increased levels of pollutants at lags 0–4 days

Pollutant	Events	All MI		STEMI		Non-STEMI	
		% change (95% CI)		% change (95% CI)		% change (95% CI)	
CO	343 914	0.2	(−1.4 to 1.9)	1.4	(−1.2 to 3.9)	−0.6	(−2.8 to 1.6)
NO_2_	405 799	2.4	(0.3 to 4.5)	1.4	(−1.8 to 4.6)	3.1	(0.3 to 6.0)
O_3_	410 341	−1.4	(−3.1 to 0.4)	−2.8	(−5.5 to −0.1)	−0.3	(−2.6 to 2.0)
PM_10_	365 151	−0.6	(−1.9 to 0.7)	−0.5	(−2.5 to 1.5)	−0.6	(−2.3 to 1.1)
PM_2.5_	134 964	−0.4	(−2.4 to 1.6)	−2.7	(−5.6 to 0.3)	1.2	(−1.4 to 3.8)
SO_2_	380 743	1.7	(−0.1 to 3.4)	0.8	(−1.9 to 3.5)	2.3	(0.0 to 4.7)

MI, myocardial infarction; STEMI, ST-elevation MIs.

*Per cent change in risk for a 10th–90th centile change in pollutant in 2003–2009.

Data source: MINAP registry, 2003–2009.

CO, carbon monoxide; MINAP, Myocardial Ischaemia National Audit Project; NO_2_, nitrogen dioxide; PM_2.5_, particulate matter less than 2.5 μm in aerodynamic diameter; PM_10_, particulate matter less than 10 μm in aerodynamic diameter; SO_2_, sulfur dioxide.

### Hospital admissions

**Figure 1:**
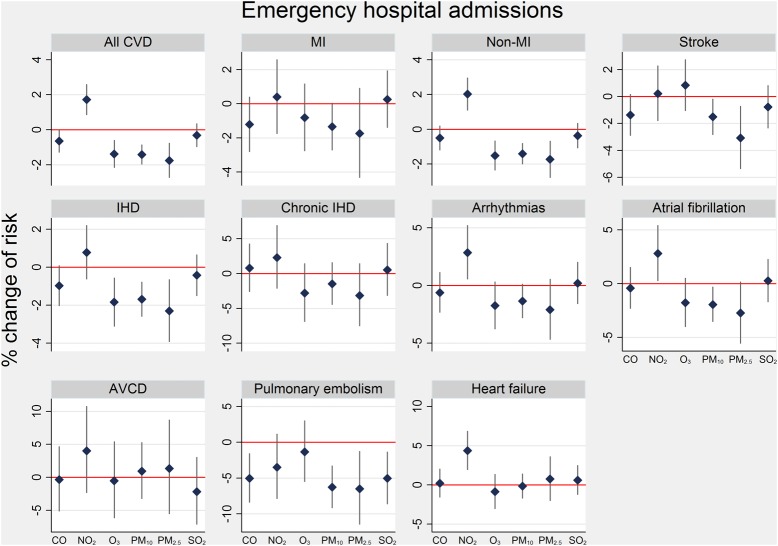
Per cent change (95% CI) in risk of emergency cardiovascular admissions for a 10th–90th centile change in pollutant at lags 0–4 days. 10th–90th centile ranges in pollutants for 2003–2008. AVCD, atrioventricular conduction disorder; MI, myocardial infarction; IHD, ischaemic heart disease. Data source: Hospital Episode Statistics database, 2003–2008.

Figure 1 presents effects on emergency hospital admissions. Only NO_2_ was associated with higher risk of admission for any of the causes: a 10th–90th centile change was associated with an increase of 1.7% (95% CI 0.9 to 2.6%) for CVD, 2.0% (1.1 to 2.9) non-MI CVD, 2.9% (0.6 to 5.2%) arrhythmias, 2.8% (0.3 to 5.4%) atrial fibrillation and 4.4% (2.0 to 6.8%) heart failure. Such NO_2_ association was significantly greater in females and those aged 70+ years for non-MI admissions and in females for arrhythmia (see online supplementary table S2). For all outcomes, PM_2.5_ or PM_10_ showed little evidence of increased risk of admissions, and indeed, in many cases, the risks were negative. Ozone was associated with reduced admissions for CVD, non-MI CVD and IHD.

### Mortality

**Figure 2:**
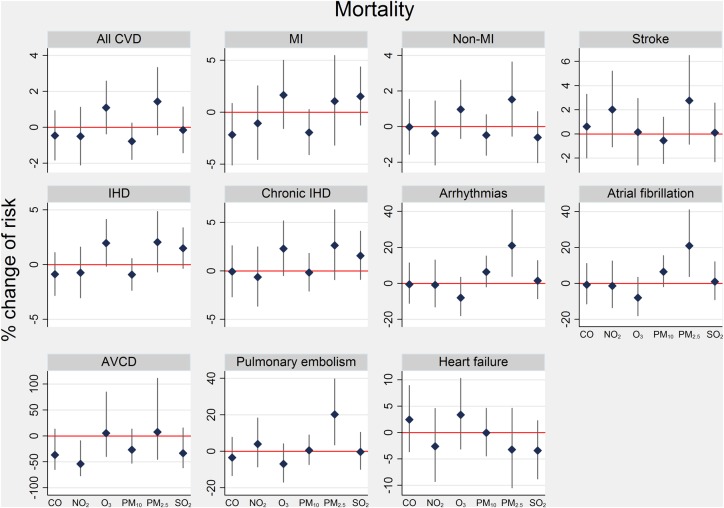
Per cent change (95% CI) in risk of cardiovascular mortality for a 10th–90th centile change in pollutant at lags 0–4 days. 10th–90th centile ranges in pollutants for 2003–2006. AVCD, atrioventricular conduction disorder; MI, myocardial infarction; IHD, ischaemic heart disease. Data source: Office of National Statistics mortality registry, 2003–2006.

Figure 2 presents effects on mortality outcomes. None were clearly associated with pollutants, with the exception of PM_2.5_ on some outcomes: a 10th–90th centile change was associated with an increase of 21% (95% CI 3.9 to 40.8%) for arrhythmias, 21% (3.9 to 41%) atrial fibrillation and 20.5% (3.5 to 39.7%) pulmonary embolism. For all CVD deaths and deaths from IHD, PM_2.5_ and O_3_ risk was significantly raised among women (see online supplementary table S3). Also, those aged 70+ years were at significantly greater risk from O_3_ exposure on all CVD and IHD deaths.

### Sensitivity analyses

The above results were robust to (i) adjustment for a second pollutant, except PM_2.5_ effects on mortality, which were reduced by 10–15% and rendered non-significant after adjustment for NO_2_; (ii) summer-specific effects for O_3_ in MINAP models; (iii) effects assessed at just lags 0–1 days, except PM_2.5_ on admissions for arrhythmias (see online supplementary figure S5); (iv) adjustment for day-of-week by matching; and (v) clustering effects by monitoring sites.

## Discussion

### Summary of findings

We observed little acute effect of ambient air pollution on STEMIs or stroke. NO_2_ was associated with admissions for CVD overall, non-MI CVD, arrhythmias including atrial fibrillation and heart failure. PM_2.5_ showed some adverse effects on all CVD deaths, arrhythmias and pulmonary embolism. In general, effects of NO_2_ on hospital admissions and PM_2.5_ on mortality were higher in those over 70 years and in females, although sex differences may also reflect differences in the age distribution.

### Comparison with other studies

Our results are mostly consistent with a previous analysis of the MINAP database, which observed a detrimental effect of pollution in 1–6 h but with little net effect at the daily level.^11^ Their findings suggest that any evident initial risk from pollution exposure may have been demonstrating a bringing forward of MI events in already vulnerable individuals (displacement by a few hours). However, our sensitivity analysis showed an increased effect of NO_2_ and SO_2_ on non-STEMI at lags 0–4 days compared with lags 0–1 days, suggesting little evidence for displacement at the daily level.

The general lack of a pollution effect on MI (especially STEMI) risk in the current study is not unexpected. Although a recent systematic review reported most air pollutants were associated with a short-term increase in MI risk,^5^ a previous review revealed less than half of the identified studies found clear evidence of raised MI risk from exposure to pollutants_._^12^ Furthermore, the studies that did not detect a detrimental effect tended to be those that had better temperature control (eg, considered lagged effects) and also were more likely to have separate validation of MI diagnosis. Another comprehensive review of CVD outcomes in relation to particulate matter indicated that results related to thrombosis/coagulation were variable due to differences in study designs, patients and biomarkers evaluated, but that adverse effects were most consistent among high-risk groups.^3^ However, we observed little effect of PM_10_ or PM_2.5_ specifically on MI events, even among high-risk individuals.

Contrary to previous studies,^13–16^ we observed little effect of pollutants on cardiovascular mortality, although some adverse PM_2.5_ effect was detected. Results from a follow-up study of Medicare patients^17^ indicated that smaller particles and their components derived from combustion sources (ie, PM_2.5_) are principally responsible for cardiovascular hospitalisations attributed to the combination of fine and coarse particles (ie, PM_10_). In our study, adverse effects of PM_2.5_ or PM_10_ on cardiovascular admissions were not apparent but strong NO_2_ effects were observed, particularly with non-MI CVD, arrhythmias including atrial fibrillation and heart failure. Although NO_2_ and PM measures are correlated (see online supplementary table S4), the strong NO_2_ effect on admissions persisted after adjustment for PM_2.5_. By contrast, the PM_2.5_ effect on mortality outcomes was somewhat reduced when NO_2_ was adjusted for. Previous work from Europe has highlighted that higher NO_2_ levels can be associated with larger PM_10_ effects on mortality.^18^ Differences between our results for mortality and hospital admissions outcomes may have been explained by a smaller set of linked mortality events compared with admissions, with possibly different patient characteristics. However, our NO_2_ effect on admissions was even greater when restricted to the subset of patients who could be successfully linked to a PM_2.5_ monitor also. In addition, PM_2.5_ appeared to increase the risk of death where the *recorded underlying* cause was an arrhythmia, including atrial fibrillation (though the proximate cause may have been a complication of such arrhythmia), and pulmonary embolism, but reduced hospitalisation for these causes. One possible explanation might be those effects are rapid and so vulnerable individuals may bypass medical presentation, as suggested by Rich *et al.*^19^ Curiously, our sensitivity analysis showed an adverse effect of PM_2.5_ at lags 0–1 days, which was then protective by lags 0–4 days.

A protective effect was also observed with O_3_ on some CVD outcomes, which has also been observed by previous studies.^12^ This effect persisted in two-pollutant models and also when MINAP events were restricted to just the summer months when O_3_ concentrations are at their highest. Such patterns may be due to its negative correlation with an unmeasured pollutant or may reflect the fact that O_3_ is highly reactive and so measurements based on outdoor monitors may not be a good proxy for personal exposure.

### Biological mechanisms

Observational and experimental studies have proposed a number of pathways to explain how air pollution may affect the cardiovascular system. The main candidate hypotheses are the disruption of the autonomic nervous system and/or an inflammatory response.^20^ There is evidence that the specific biological mechanisms that then trigger cardiovascular events include vascular dysfunction or vasoconstriction, enhanced thrombosis or coagulation potential, elevated arterial blood pressure, enhanced atherosclerosis or plaque vulnerability, and arrhythmias.^3^

Animal studies show that air pollutants affect the cardiac autonomic nervous system.^21^ Exposure of dogs to concentrated ambient particles leads to alterations in heart rate variability and thus a disturbance in cardiac autonomic control.^22^ However, in human experimental studies, heart rate variability or heart rhythm has not been found to be associated with diesel exhaust fumes,^23^ ^24^ which is discrepant with evidence from observational studies.^25^

Although it is likely that air pollution affects cardiovascular health via multiple mechanisms, the lack of pollution effects on STEMIs and stroke, but the stronger associations with selected non-MI outcomes in the current study, suggests that pollution effects on cardiovascular health may *in part* be mediated by non-thrombotic pathways. However, thrombogenic mechanisms may still operate and are the most likely explanation for the observed associations with pulmonary embolism, for example.

Besides, we cannot exclude the possibility of the consequences of systemic inflammation occurring over longer time periods, which may then predispose vulnerable individuals to future cardiovascular events.

### Strengths and limitations

A major strength of this study is the national coverage of a wide range of CVD outcomes. A previous study using a similarly large population in 204 US counties only considered elderly patients in urban areas.^26^ In our study, all acute hospitals in England and Wales are included on the MINAP database and thus should not under-represent any subgroups of the population, including those living in rural areas, except with PM_2.5_ (discussed below). An additional strength is the specific case definitions of MI in the MINAP database using large amounts of clinical information.

There are some limitations. First, the consistency of results on MI using MINAP and HES data is to be expected as there will be considerable overlap between the two databases. Also, no information is available on those MIs that result in death before admission. It is therefore possible that pollution effects may be missed if they result in fatal heart attacks before coming to medical attention, although there is no reason to suspect such a specific effect that is not observed in MI patients who reach hospital. More extensive analysis of long-term exposure effects on mortality among MINAP patients has been reported separately.^27^ Second, we used fixed monitoring sites to represent air pollution and so may not accurately reflect personal exposure. However, we excluded roadside and kerbside monitors and correlations between other stations were generally high within 50 km of each other. A 50 km limit ensured that a high number of CVD events were successfully linked to exposure (over 80% for all pollutants except PM_2.5_). It is possible that some exposure misclassification may have contributed to the largely null results and that restricting analyses to events substantially nearer the monitoring sites may have revealed stronger associations. However, a previous analysis of MINAP using pollution and event data restricted to within the major conurbations also observed little pollution effect over a 3-day period.^11^ Third, the number of national PM_2.5_ monitoring sites is limited in the UK and most of the sites are located in urban residential areas. Thus, our PM_2.5_ effects may not be representative of those living in other settings. Ultrafine particles, black carbon or NO_x_ may be better indicators of primary combustion particles compared with PM_10_ and PM_2.5_ from urban background stations, which also reflect secondary PM and road dust. However, there is no such national monitoring network in the UK. Here we note that the effects of black carbon may not be dissimilar to PM_2.5_ or PM_10_ when considered in terms of a percentile change, as previously reported.^28^ Finally, the meaning of specific pollutant concentration is not simple to interpret as each typically acts as an indicator of a mixture as well a specific pollutant, with the latter meaning changing in two-pollutant models.

## Conclusions

This study found no clear evidence for pollution effects on STEMIs and stroke, which ultimately represent thrombogenic processes, though it did for pulmonary embolism. The strongest associations with air pollution were observed with selected non-MI outcomes. Experimental studies with tightly controlled pollution mixes, in addition to large-scale observational studies such as this, are needed to help elucidate mechanistic pathways further. Elderly people and hospital patients with chronic IHD or arrhythmias are observed to be at particular risk, which could help inform intervention strategies.

**Key messages**

- **What is already known on this subject?**
- High levels of some air pollutants are associated with increased risk of cardiovascular health outcomes.
- The likely mechanisms of pollution-related cardiovascular disease are uncertain.
- **What this study adds?**
- Short-term exposure to NO_2_ and PM_2.5_ has adverse effects on selected non-MI outcomes, especially arrhythmia.
- This study found no clear evidence for pollution effects on STEMIs and stroke, which ultimately represent thrombogenic processes, though it did for pulmonary embolism.
- Elderly people and hospital patients with chronic IHD or arrhythmias are observed to be at particular risk of MI.
- **How might this impact on clinical practice?**
- This study provides further evidence on current understanding of pathophysiological mechanisms by examining the strength and specificity of acute relationships between ambient air pollution and a range of cardiovascular disease events.
- Subgroup analyses using detailed patient information of MINAP database might help inform intervention strategies.

## Supplementary Material

Web supplement
