# Supplementary material for: Short-term effects of air pollution on a range of cardiovascular events in England and Wales: case-crossover analysis of the MINAP database, hospital admissions and mortality
Source: Heart. 2014 Jun 4;100(14):1093–8. doi: 10.1136/heartjnl-2013-304963 (PMC4078678; doi:10.1136/heartjnl-2013-304963)
Supplement: Web supplement [file heartjnl-2013-304963-s1.pdf]

Online supplements

Contents list:

Table S1. Patient information of all STEMI (N=189,410) and non-STEMI (N=262,933) events in MINAP 2003-2009.

Table S2. Percentage change (Δ%) in risk of CVD emergency hospital admissions for a 10th-90th percentile range change in pollutant (in 2003-2008) at lags 0-4days. Models included the presented pollutant effects, matched with lunar month and adjusted for day of week and temperature (lag0-1, 2-7, 8-14). Stratified by sex and age group. Data source: HES database 2003-2008.

Table S3. Percentage change (Δ%) in risk of CVD mortality for a 10th-90th percentile change in pollutant (in 2003-2006) at lags 0-4days. Models included the presented pollutant effects, matched with lunar month and adjusted for day of week and temperature (lag0-1, 2-7, 8-14). Stratified by sex and age group. Data source: ONS mortality registry 2003-2006.

Table S4 Within-stratum correlation between pollutants: [a] HES analysis and [b] mortality analysis

Figure S1. AURN monitoring sites for air pollution (red points, except roadside and curbside) and BADC sites for temperature (blue points) in 2003-2009. Gray dots show MINAP patient's residential addresses.

Figure S2. Correlations and mean differences of daily pollution and temperature values by distance. Points represent agreement between all possible combinations of 2 monitors. Figures show agreement separately by season (April-September and October-March) and by distances (within 500km and then just within 50km). $PM_{2.5}$ is not shown due to small number of monitoring stations.

Figure S3. Effects of NO_2 at lags 0-4days on [a] STEMI and [b] non-STEMI diagnosis by risk factors. Column on extreme right shows P-value from interactions model.

Figure S4. Effects of SO_2 at lags 0-4days on [a] STEMI and [b] non-STEMI diagnosis by risk factors. Column on extreme right shows P-value from interactions model.

Figure S5. Percentage change (95%CI) in risk of cardiovascular events for a 10th-90th percentile range change in pollutant at lags 0-1 days and lags 0-4 days. 10th-90th percentile ranges in pollutant vary in databases: [a] MINAP 2003-2009, [b] HES 2003-2008, and [c] ONS mortality 2003-2006. AVCD, MI, and IHD represent Atrio-venticular conduction disorder, myocardial infarction, and ischaemic heart disease respectively.

Table S1. Patient information of all STEMI (N=189,410) and non-STEMI (N=262,933) events in MINAP 2003-2009.

Patient characteristic	Response	STEMI events		Non-STEMI events	
		No.	%[a]	No.	%[a]
Sex	Male	131226	69.3	161691	61.5
	Female	57360	30.3	100626	38.3
Age	< 70 years	107878	57.0	97091	36.9
	70+ years	78753	41.6	158750	60.4
Patient status	Alive	137596	72.6	161041	61.2
	Dead	45475	24.0	95590	36.4
Smoking status	Non-smoker	35772	18.9	59837	22.8
	Ex/current smoker	111346	58.8	141352	53.8
Previous MI	No	137072	72.4	160689	61.1
	Yes	27228	14.4	79198	30.1
Previous angina	No	130643	69.0	142527	54.2
	Yes	30847	16.3	94426	35.9
Previous lipids	No	109993	58.1	151828	57.7
	Yes	45025	23.8	77651	29.5
Previous hypertension	No	93595	49.4	113495	43.2
	Yes	68981	36.4	124711	47.4
Presence of peripheral vascular disease	No	150031	79.2	212690	80.9
	Yes	5279	2.8	14018	5.3
History of cerebrovascular disease	No	147111	77.7	204865	77.9
	Yes	9439	5.0	24851	9.5
Asthma or COPD	No	135437	71.5	186348	70.9
	Yes	18878	10.0	39145	14.9
Chronic renal failure	No	153642	81.1	215106	81.8
	Yes	3694	2.0	15035	5.7
Congestive cardiac failure	No	153011	80.8	208825	79.4
	Yes	4310	2.3	21516	8.2
Previous percutaneous coronary intervention	No	148977	78.7	211058	80.3
	Yes	8985	4.7	20278	7.7
Previous coronary artery bypass grafting	No	154157	81.4	214754	81.7
	Yes	4339	2.3	17916	6.8
Diabetes	No	141976	75.0	185115	70.4
	Yes	22599	11.9	54620	20.8
Angiotensin converting enzyme inhibitor in regular use before admission	No	75638	39.9	92972	35.4
	Yes	31805	16.8	69993	26.6
Betablocker in regular use before admission	No	79949	42.2	105791	40.2
	Yes	27570	14.6	57256	21.8
Statin in regular use before admission	No	72129	38.1	81568	31.0
	Yes	37477	19.8	84271	32.1
Clopidogrel in regular use before admission	No	73856	39.0	103232	39.3
	Yes	15556	8.2	29331	11.2
Aspirin/anti-platelet in regular use before admission	No or started now	144525	76.3	159265	60.6
	Yes	28694	15.1	80907	30.8

COPD, chronic obstructive pulmonary disease; MINAP, myocardial ischaemia national audit project; STEMI, ST-elevation myocardial infarction.
[a] % of all events, which does not add to 100 where missing responses were present

Table S2. Percentage change (Δ%) in risk of CVD emergency hospital admissions for a 10th-90th percentile range change in pollutant (in 2003-2008) at lags 0-4days. Models included the presented pollutant effects, matched with lunar month and adjusted for day of week [a] and temperature (lag0-1, 2-7, 8-14). Stratified by sex and age group. Data source: HES database 2003-2008.

Outcome	Pollutant	All			By sex					By age group				
					Male		Female		*P*	<70 years old		70+ years old		*P*
		N. of events	Δ%	(95%CI)	Δ%	(95%CI)	Δ%	(95%CI)		Δ%	(95%CI)	Δ%	(95%CI)	
CVD	CO	2,315,102	-0.6	(-1.3, 0.0)	-0.7	(-1.5, 0.1)	0.6	(-0.3, 1.5)	0.19	-1.5	(-2.4, -0.5)	0.0	(-0.8, 0.8)	0.02
	NO_2	2,634,065	1.7	(0.9, 2.6)	1.7	(0.6, 2.9)	1.7	(0.5, 2.9)	0.03	0.2	(-1.0, 1.4)	2.9	(1.8, 4.0)	<0.01
	O_3	2,663,067	-1.4	(-2.1, -0.6)	-1.9	(-2.8, -0.9)	-0.8	(-1.9, 0.2)	0.28	-1.5	(-2.5, -0.4)	-1.3	(-2.2, -0.4)	<0.01
	PM_{10}	2,422,697	-1.4	(-1.9, -0.9)	-1.5	(-2.2, -0.8)	-1.3	(-2.1, -0.6)	0.70	-1.9	(-2.7, -1.1)	-1.1	(-1.8, -0.4)	0.17
	$PM_{2.5}$	706,957	-1.7	(-2.7, -0.8)	-2.0	(-3.2, -0.7)	-1.5	(-2.8, -0.1)	0.05	2.2	(-3.5, -0.8)	-1.4	(-2.7, -0.2)	0.64
	SO_2	2,499,129	-0.3	(-1.0, 0.3)	-0.6	(-1.4, 0.3)	0.0	(-0.9, 0.9)	0.50	1.2	(-2.2, -0.3)	0.4	(-0.5, 1.2)	0.03
Non-MI	CO	1,978,859	-0.5	(-1.2, 0.2)	-0.9	(-1.8, 0.0)	0.0	(-1.0, 0.9)	0.13	-1.5	(-2.5, -0.5)	0.2	(-0.7, 1.1)	0.03
	NO_2	2,253,105	2.0	(1.1, 2.9)	1.6	(0.4, 2.8)	2.5	(1.2, 3.8)	0.02	0.5	(-0.8, 1.9)	3.2	(2.0, 4.3)	0.02
	O_3	2,277,016	-1.5	(-2.3, -0.7)	-1.6	(-2.7, -0.5)	-1.4	(-2.5, -0.3)	0.49	-1.5	(-2.6, -0.3)	-1.5	(-2.5, -0.5)	<0.01
	PM_{10}	2,072,273	-1.4	(-2.0, -0.8)	-1.6	(-2.4, -0.8)	-1.2	(-2.0, -0.4)	0.71	-1.9	(-2.7, -1.0)	-1.1	(-1.8, -0.3)	0.35
	$PM_{2.5}$	614,630	-1.7	(-2.8, -0.7)	-2.2	(-3.5, -0.8)	-1.2	(-2.6, 0.2)	0.10	-2.1	(-3.6, -0.6)	-1.4	(-2.7, -0.1)	0.65
	SO_2	2,137,313	-0.4	(-1.1, 0.3)	-0.8	(-1.7, 0.2)	0.1	(-0.9, 1.1)	0.22	-1.3	(-2.3, -0.2)	0.3	(-0.6, 1.2)	0.06
Stroke	CO	368,081	-1.4	(-2.9, 0.2)	-0.1	(-2.2, 2.1)	-2.5	(-4.5, -0.5)	0.20	-4.8	(-7.4, -2.3)	0.2	(-1.6, 2.0)	0.01
	NO_2	421,340	0.2	(-1.8, 2.3)	0.9	(-1.9, 3.7)	-0.4	(-3.0, 2.4)	0.16	-3.1	(-6.4, 0.3)	1.8	(-0.6, 4.2)	<0.01
	O_3	426,940	0.8	(-1.0, 2.7)	2.0	(-0.5, 4.5)	-0.2	(-2.5, 2.2)	0.25	-1.5	(-4.4, 1.6)	1.8	(-0.3, 4.0)	0.10
	PM_{10}	385,412	-1.5	(-2.8, -0.2)	-1.0	(-2.8, 0.9)	-2.0	(-3.7, -0.2)	0.79	-2.8	(-5.0, -0.6)	-0.9	(-2.5, 0.6)	0.10
	$PM_{2.5}$	114,924	-3.1	(-5.4, -0.7)	-2.7	(-5.8, 0.5)	-3.4	(-6.4, -0.3)	0.26	-4.4	(-8.1, -0.5)	-2.5	(-5.1, 0.2)	0.71
	SO_2	397,873	-0.8	(-2.3, 0.8)	-0.6	(-2.8, 1.7)	-1.0	(-3.1, 1.2)	0.96	-2.2	(-4.9, 0.5)	-0.1	(-2, 1.8)	0.13
IHD [b]	CO	786,938	-1.0	(-2.0, 0.1)	-0.3	(-1.6, 1.0)	-2.0	(-3.6, -0.4)	0.37	-1.4	(-2.8, 0.1)	-0.6	(-2.0, 0.8)	0.01
	NO_2	887,432	0.8	(-0.6, 2.2)	1.5	(-0.2, 3.3)	-0.4	(-2.5, 1.8)	0.20	-1.3	(-3.1, 0.7)	2.7	(0.8, 4.7)	0.09
	O_3	898,275	-1.8	(-3.1, -0.6)	-2.7	(-4.2, -1.1)	-0.6	(-2.4, 1.3)	0.12	-0.9	(-2.6, 0.8)	-2.6	(-4.2, -1.0)	0.04
	PM_{10}	820,198	-1.7	(-2.6, -0.8)	-1.2	(-2.3, 0.0)	-2.5	(-3.8, -1.1)	0.65	-2.4	(-3.6, -1.1)	-1.1	(-2.3, 0.2)	0.02
	$PM_{2.5}$	234,692	-2.3	(-3.9, -0.7)	-2.0	(-4.0, 0.0)	-2.8	(-5.2, -0.3)	0.84	-1.8	(-4.0, 0.4)	-2.8	(-4.9, -0.6)	0.41
	SO_2	845,927	-0.4	(-1.5, 0.6)	-0.6	(-1.9, 0.8)	-0.2	(-1.9, 1.4)	0.38	-1.2	(-2.7, 0.3)	0.3	(-1.1, 1.8)	0.71
MI	CO	336,243	-1.2	(-2.8, 0.4)	0.2	(-1.7, 2.2)	-3.7	(-6.1, -1.2)	0.01	-1.3	(-3.6, 1.1)	-1.2	(-3.2, 0.9)	0.40
	NO_2	380,960	0.4	(-1.7, 2.6)	2.1	(-0.6, 4.8)	-2.4	(-5.6, 0.9)	0.32	-2.1	(-5.1, 1.0)	2.4	(-0.4, 5.3)	0.13
	O_3	386,051	-0.8	(-2.7, 1.2)	-2.7	(-5.0, -0.4)	2.5	(-0.5, 5.5)	0.06	-1.4	(-4.1, 1.3)	-0.4	(-2.8, 2.1)	0.87
	PM_{10}	350,424	-1.3	(-2.7, 0.0)	-1.0	(-2.7, 0.7)	-2.0	(-4.1, 0.2)	0.93	-2.0	(-4.0, 0.0)	-0.8	(-2.6, 1.0)	0.30
	$PM_{2.5}$	92,327	-1.7	(-4.3, 0.9)	-1.3	(-4.4, 1.9)	-2.6	(-6.5, 1.5)	0.26	-2.3	(-5.9, 1.4)	-1.3	(-4.6, 2.1)	0.58
	SO_2	361,816	0.3	(-1.4, 1.9)	0.2	(-1.8, 2.3)	0.3	(-2.3, 3.0)	0.73	-1.0	(-3.4, 1.5)	1.2	(-0.9, 3.4)	0.55
Chronic IHD	CO	72,529	0.8	(-2.6, 4.2)	0.3	(-3.6, 4.5)	1.6	(-3.9, 7.5)	0.89	-2.0	(-6.3, 2.5)	4.1	(-0.8, 9.2)	0.25

	NO_2	81,169	2.3	(-2.1, 6.9)	1.3	(-3.8, 6.8)	4.3	(-3, 12.1)	0.70	-2.2	(-7.6, 3.6)	8.0	(1.5, 15.0)	0.06
	O_3	81,206	-2.8	(-6.9, 1.4)	-2.6	(-7.3, 2.3)	-3.2	(-9.4, 3.5)	0.49	-1.8	(-7.0, 3.6)	-4.0	(-9.3, 1.7)	0.43
	PM_{10}	76,402	-1.5	(-4.4, 1.6)	-4.2	(-7.6, -0.6)	4.0	(-1.0, 9.3)	0.02	-4.1	(-7.8, -0.2)	1.7	(-2.6, 6.2)	0.27
	$PM_{2.5}$	30,441	-3.1	(-7.5, 1.4)	-4.0	(-9.0, 1.4)	-1.4	(-8.6, 6.2)	0.76	-2.0	(-7.6, 4.0)	-4.6	(-10.5, 1.8)	0.72
	SO_2	77,536	0.5	(-3.1, 4.3)	-1.3	(-5.7, 3.2)	4.0	(-2.1, 10.5)	0.26	-1.3	(-6.1, 3.7)	2.6	(-2.7, 8.2)	0.50
Arrythmias [c]	CO	305,027	-0.6	(-2.3, 1.1)	-0.2	(-2.5, 2.2)	-1.0	(-3.3, 1.3)	0.12	-0.7	(-3.1, 1.8)	-0.6	(-2.8, 1.7)	0.49
	NO_2	349,238	2.9	(0.6, 5.2)	2.1	(-1.0, 5.2)	3.6	(0.5, 6.8)	<0.01	1.8	(-1.4, 5.1)	3.7	(0.7, 6.8)	0.39
	O_3	352,775	-1.7	(-3.8, 0.3)	-2.4	(-5.0, 0.3)	-1.1	(-3.7, 1.6)	0.02	-1.2	(-3.9, 1.6)	-2.2	(-4.7, 0.4)	0.46
	PM_{10}	319,992	-1.4	(-2.8, 0.1)	-1.7	(-3.6, 0.4)	-1.1	(-3.0, 0.9)	0.84	-0.3	(-2.4, 1.8)	-2.2	(-4.1, -0.3)	0.16
	$PM_{2.5}$	93,302	-2.1	(-4.7, 0.5)	-4.0	(-7.4, -0.5)	-0.2	(-3.7, 3.4)	0.04	-3.1	(-6.6, 0.6)	-1.3	(-4.6, 2.1)	0.77
	SO_2	329,941	0.2	(-1.6, 2.0)	0.7	(-1.8, 3.2)	-0.2	(-2.6, 2.2)	0.62	1.3	(-1.3, 3.9)	-0.7	(-3.0, 1.7)	0.40
Atrial Fibrillation	CO	249,702	-0.4	(-2.3, 1.5)	0.3	(-2.3, 2.9)	-1.1	(-3.6, 1.5)	0.11	-0.1	(-2.9, 2.8)	-0.7	(-3.0, 1.8)	0.79
	NO_2	285,898	2.8	(0.3, 5.4)	2.8	(-0.7, 6.3)	2.8	(-0.5, 6.3)	0.02	1.2	(-2.5, 5.0)	3.9	(0.7, 7.2)	0.30
	O_3	288,617	-1.8	(-4.0, 0.5)	-3.2	(-6.0, -0.2)	-0.5	(-3.3, 2.5)	0.05	-0.8	(-3.9, 2.5)	-2.5	(-5.2, 0.3)	0.19
	PM_{10}	262,106	-1.9	(-3.5, -0.3)	-2.6	(-4.8, -0.4)	-1.3	(-3.5, 0.9)	0.67	-1.4	(-3.7, 1.0)	-2.3	(-4.3, -0.3)	0.31
	$PM_{2.5}$	76,548	-2.7	(-5.6, 0.1)	-4.8	(-8.5, -0.9)	-0.8	(-4.6, 3.1)	0.04	-4.4	(-8.4, -0.2)	-1.6	(-5.1, 2.0)	0.45
	SO_2	269,880	0.3	(-1.7, 2.2)	0.7	(-2.0, 3.5)	-0.2	(-2.8, 2.5)	0.68	1.7	(-1.3, 4.7)	-0.7	(-3.2, 1.8)	0.32
AVCD	CO	38,325	-0.4	(-5.1, 4.6)	-0.2	(-6.2, 6.2)	-0.6	(-7.6, 6.9)	0.92	3.4	(-5.5, 13.3)	-1.7	(-7.1, 3.9)	0.33
	NO_2	43,973	4.0	(-2.3, 10.7)	0.9	(-6.8, 9.2)	8.7	(-1, 19.2)	0.58	-1.8	(-12.5, 10.2)	6.2	(-1.2, 14.1)	0.78
	O_3	44,445	0.5	(-6.1, 5.4)	-0.9	(-7.7, 6.3)	0.0	(-7.8, 8.4)	0.65	-0.1	(-9.6, 10.5)	-0.7	(-6.9, 5.9)	0.91
	PM_{10}	40,083	0.9	(-3.2, 5.3)	-1.1	(-6.2, 4.3)	3.9	(-2.4, 10.5)	0.86	-1.1	(-8.4, 6.9)	1.6	(-3.1, 6.6)	0.49
	$PM_{2.5}$	13,646	1.3	(-5.5, 8.7)	-4.4	(-12.4, 4.3)	10.1	(-0.5, 22)	0.24	5.6	(-7.0, 19.8)	-0.2	(-7.8, 8.0)	0.29
	SO_2	41,405	-2.2	(-7.1, 3.0)	-3.6	(-9.8, 3.0)	-0.2	(-7.7, 8.0)	0.25	1.3	(-7.8, 11.3)	-3.6	(-9.3, 2.4)	0.09
Pulmonary embolism	CO	70,168	-5.0	(- 8.3, - 1.6)	-7.0	(-11.6, -2.1)	-3.4	(-7.8, 1.2)	0.76	-8.5	(-12.7, -4.2)	-0.6	(-5.5, 4.6)	0.03
	NO_2	81,525	-3.5	(- 7.9, 1.1)	-5.3	(-11.3, 1.2)	-2.0	(-7.7, 4.1)	0.86	-7.4	(-12.8, -1.6)	1.6	(-4.9, 8.7)	<0.01
	O_3	82,231	-1.3	(- 5.5, 3.0)	-0.8	(-6.4, 5.1)	-1.7	(-6.9, 3.7)	0.29	2.4	(-2.9, 8.1)	-5.8	(-11.2, -0.1)	0.09
	PM_{10}	74,092	-6.3	(- 9.1, - 3.3)	-8.1	(-12.1, -4.0)	-4.7	(-8.5, -0.7)	0.79	-8.1	(-11.7, -4.3)	-3.9	(-8.1, 0.5)	0.05
	$PM_{2.5}$	22,391	-6.5	(-11.4, - 1.3)	-3.2	(-10.2, 4.3)	-9.3	(-15.4, -2.6)	0.69	-13	(-18.9, -6.8)	2.2	(-5.3, 10.2)	0.01
	SO_2	76,730	-5.0	(- 8.6, - 1.4)	-7.0	(-12, -1.8)	-3.4	(-8.1, 1.6)	0.49	-9.1	(-13.4, -4.5)	0.6	(-4.9, 6.4)	0.03
Heart failure	CO	272,763	0.2	(- 1.5, 2.0)	-1.1	(-3.4, 1.4)	1.5	(-0.9, 4.0)	0.14	2	(-1.8, 5.9)	-0.2	(-2.1, 1.8)	0.47
	NO_2	308,543	4.4	(2.0, 6.8)	4.5	(1.2, 7.9)	4.2	(1.0, 7.6)	0.04	5.9	(0.8, 11.2)	4.0	(1.4, 6.7)	0.23
	O_3	312,332	-0.9	(- 3.0, 1.3)	-0.7	(-3.5, 2.2)	-1.0	(-3.8, 1.9)	0.81	-1.1	(-5.3, 3.3)	-0.8	(-3.1, 1.6)	0.27
	PM_{10}	284,550	-0.2	(- 1.7, 1.4)	-0.7	(-2.8, 1.4)	0.4	(-1.6, 2.6)	0.57	0.7	(-2.6, 4.0)	-0.3	(-2.0, 1.3)	0.71
	$PM_{2.5}$	82,879	0.8	(- 2.0, 3.6)	-0.8	(-4.4, 3.0)	2.3	(-1.4, 6.2)	0.74	3	(-2.7, 9.1)	0.2	(-2.8, 3.3)	0.30
	SO_2	293,763	0.6	(- 1.2, 2.5)	-0.3	(-2.8, 2.2)	1.5	(-1.0, 4.1)	0.10	-1.9	(-5.7, 2.1)	1.2	(-0.8, 3.3)	0.18

AVCD, atrio-ventricular conduction disorders; HES, hospital episode statistics; IHD, ischaemic heart disease; MI, myocardial infarction.
P value by 2-sided Wald test for overall interaction terms
[a] For all CVD and non-MI admissions, day of week is matched as well as lunar month because of limitation in computing memory.
[b] including MI and chronic IHD
[c] excluding conduction disorders and bradycardias, including atrial fibrillation

Table S3. Percentage change (Δ%) in risk of CVD mortality for a 10th-90th percentile change in pollutant (in 2003-2006) at lags 0-4days. Stratified by sex and age group. Data source: ONS mortality registry.

Outcome	Pollut-ant	All			By sex					By age group				
		N. of events			Male		Female		*P*	<70 years old		70+ years old		*P*
			Δ%	(95%CI)	Δ%	(95%CI)	Δ%	(95%CI)		Δ%	(95%CI)	Δ%	(95%CI)	
CVD	CO	624,839	-0.5	(-1.8, 0.9)	-0.3	(-2.2, 1.7)	-0.7	(-2.5, 1.2)	0.07	-2.3	(-5.3, 0.8)	-0.1	(-1.6, 1.4)	0.69
	NO_2	683,381	-0.5	(-2.1, 1.1)	0.1	(-2.1, 2.4)	-1.1	(-3.2, 1.1)	0.53	-1.6	(-5.1, 2)	-0.3	(-2, 1.5)	0.01
	O_3	692,886	1.1	(-0.4, 2.6)	-0.2	(-2.1, 1.7)	2.3	(0.4, 4.2)	0.04	-1.2	(-4.1, 1.9)	1.6	(0, 3.2)	0.02
	PM_{10}	633,383	-0.8	(-1.8, 0.2)	-0.8	(-2.2, 0.6)	-0.7	(-2.1, 0.6)	0.02	-0.9	(-3.1, 1.4)	-0.8	(-1.9, 0.3)	0.02
	$PM_{2.5}$	158,287	1.4	(-0.4, 3.3)	-1.0	(-3.5, 1.5)	3.7	(1.3, 6.3)	0.05	-1.7	(-5.5, 2.3)	2.1	(0.1, 4.2)	0.11
	SO_2	660,409	-0.2	(-1.4, 1.1)	0.1	(-1.7, 1.9)	-0.4	(-2.1, 1.4)	0.81	-1.6	(-4.4, 1.3)	0.2	(-1.2, 1.6)	0.70
Non-MI	CO	624,839	0	(-1.6, 1.5)	0.2	(-2, 2.4)	-0.2	(-2.2, 1.9)	0.07	-1.7	(-5.2, 1.9)	0.3	(-1.4, 2)	0.93
	NO_2	683,381	-0.4	(-2.2, 1.4)	0.3	(-2.2, 2.9)	-1.0	(-3.3, 1.4)	0.80	-2.8	(-6.7, 1.4)	0.1	(-1.9, 2.1)	0.27
	O_3	692,886	1.0	(-0.7, 2.6)	0	(-2.2, 2.2)	1.8	(-0.2, 3.9)	0.09	-2.1	(-5.4, 1.4)	1.6	(-0.2, 3.3)	0.02
	PM_{10}	633,383	-0.5	(-1.6, 0.7)	-0.6	(-2.2, 1.1)	-0.4	(-1.9, 1.1)	0.04	-1.6	(-4.2, 1)	-0.2	(-1.5, 1)	0.03
	$PM_{2.5}$	158,287	1.5	(-0.5, 3.6)	-0.9	(-3.6, 2)	3.6	(0.9, 6.4)	0.09	-1.1	(-5.5, 3.5)	2.1	(-0.1, 4.3)	0.29
	SO_2	660,409	-0.6	(-2.0, 0.8)	-0.7	(-2.8, 1.4)	-0.5	(-2.4, 1.4)	0.56	-3.3	(-6.5, 0.1)	-0.1	(-1.6, 1.5)	0.36
Stroke	CO	173,406	0.6	(-2.0, 3.3)	0.3	(-3.7, 4.5)	0.8	(-2.5, 4.1)	0.18	0.6	(-6.4, 8.2)	0.6	(-2.2, 3.4)	0.41
	NO_2	189,521	2.0	(-1.1, 5.2)	3.0	(-1.8, 7.9)	1.4	(-2.4, 5.4)	0.26	-1.7	(-9.6, 6.9)	2.4	(-0.8, 5.8)	0.14
	O_3	192,584	0.1	(-2.6, 2.9)	0.3	(-3.6, 4.4)	0	(-3.2, 3.4)	0.83	-2.7	(-9.4, 4.5)	0.6	(-2.3, 3.5)	0.39
	PM_{10}	183,037	-0.5	(-2.4, 1.4)	-1.1	(-4, 1.9)	-0.2	(-2.6, 2.2)	0.04	-1.5	(-6.5, 3.9)	-0.4	(-2.4, 1.7)	0.82
	$PM_{2.5}$	42,458	2.8	(-0.8, 6.5)	1.8	(-3.5, 7.4)	3.4	(-1, 7.9)	0.87	6.7	(-2.7, 17)	2.3	(-1.5, 6.2)	0.13
	SO_2	183,037	0.1	(-2.3, 2.6)	-1.9	(-5.5, 1.9)	1.4	(-1.7, 4.5)	0.16	-2.8	(-9.2, 4.2)	0.5	(-2.1, 3.1)	0.61
IHD [a]	CO	301,937	-0.9	(-2.8, 1.1)	-1.1	(-3.6, 1.5)	-0.6	(-3.4, 2.3)	0.22	-2.7	(-6.6, 1.3)	-0.4	(-2.5, 1.9)	0.59
	NO_2	329,033	-0.7	(-3.0, 1.6)	-0.8	(-3.8, 2.2)	-0.6	(-3.9, 2.8)	0.31	-1.1	(-5.7, 3.6)	-0.7	(-3.2, 1.9)	0.02
	O_3	333,324	2.0	(-0.1, 4.1)	0.8	(-1.8, 3.5)	3.4	(0.5, 6.4)	0.04	1.0	(-3, 5.1)	2.3	(0, 4.7)	0.09
	PM_{10}	306,554	-0.9	(-2.3, 0.5)	-0.6	(-2.4, 1.3)	-1.3	(-3.3, 0.8)	0.08	-0.3	(-3.1, 2.7)	-1.1	(-2.7, 0.5)	0.05
	$PM_{2.5}$	73,748	2.1	(-0.7, 4.8)	-0.8	(-4.2, 2.6)	5.8	(1.9, 9.9)	0.02	-2.6	(-7.7, 2.8)	3.2	(0.2, 6.3)	0.07
	SO_2	318,383	1.5	(-0.3, 3.4)	2.4	(0, 4.9)	0.4	(-2.3, 3.1)	0.22	0.8	(-3, 4.6)	1.7	(-0.3, 3.8)	0.83
MI	CO	127,779	-2.2	(-5.1, 0.8)	-1.8	(-5.6, 2.1)	-2.6	(-6.9, 1.8)	0.67	-4.2	(-9.8, 1.9)	-1.6	(-4.9, 1.8)	0.84
	NO_2	138,049	-1.1	(-4.6, 2.5)	-0.8	(-5.3, 3.9)	-1.4	(-6.4, 3.8)	0.44	1.7	(-5.3, 9.1)	-1.9	(-5.7, 2.1)	0.03
	O_3	139,710	1.7	(-1.6, 5.0)	-0.4	(-4.3, 3.7)	4.3	(-0.2, 9.1)	0.06	1.8	(-4.2, 8.2)	1.7	(-1.8, 5.4)	0.07
	PM_{10}	130,030	-1.9	(-4.1, 0.2)	-1.6	(-4.4, 1.2)	-2.4	(-5.4, 0.8)	0.12	1.3	(-3, 5.9)	-2.9	(-5.3, -0.5)	0.21
	$PM_{2.5}$	29,508	1.1	(-3.2, 5.5)	-1.2	(-6.4, 4.3)	3.9	(-2.1, 10.4)	0.26	-3.2	(-10.9, 5.2)	2.1	(-2.6, 7)	0.48
	SO_2	134,319	1.5	(-1.2, 4.4)	2.6	(-1.1, 6.4)	0.1	(-3.9, 4.3)	0.07	3.0	(-2.6, 8.9)	1.1	(-2, 4.3)	0.71
Chronic IHD	CO	172,705	-0.1	(-2.7, 2.6)	-0.9	(-4.2, 2.6)	0.9	(-2.9, 4.8)	0.25	-1.8	(-7, 3.7)	0.4	(-2.5, 3.4)	0.82
	NO_2	189,408	-0.6	(-3.6, 2.5)	-0.9	(-4.8, 3.1)	-0.3	(-4.6, 4.2)	0.67	-3.5	(-9.4, 2.8)	0	(-3.3, 3.5)	0.40

	O_3	192,013	2.3	(-0.5, 5.1)	1.8	(-1.7, 5.3)	2.9	(-0.9, 6.9)	0.06	0.3	(-4.9, 5.8)	2.8	(-0.2, 6)	0.34
	PM_{10}	175,061	-0.2	(-2.1, 1.8)	0.2	(-2.3, 2.8)	-0.6	(-3.3, 2.2)	0.39	-1.6	(-5.4, 2.4)	0.2	(-2, 2.3)	0.08
	$PM_{2.5}$	43,928	2.6	(-0.9, 6.3)	-0.7	(-5, 3.9)	6.9	(1.8, 12.2)	0.01	-2.1	(-8.7, 4.9)	3.8	(-0.1, 7.9)	0.06
	SO_2	182,550	1.6	(-0.6, 4.1)	2.4	(-0.9, 5.7)	0.6	(-2.9, 4.3)	0.64	-1.1	(-6.1, 4.2)	2.3	(-0.5, 5.1)	0.63
Arrhythmias [b]	CO	9,757	-0.5	(-11.0, 11.3)	4.1	(-14.2, 26.4)	-2.7	(-14.7, 11)	0.22	-58.9	(-76, -29.6)	3.1	(-8, 15.6)	<0.01
	NO_2	10,631	-0.9	(-13.0, 13.0)	7.4	(-14.2, 34.4)	-4.3	(-17.8, 11.5)	0.54	-65.7	(-81.4, -36.6)	3.3	(-9.6, 18)	<0.01
	O_3	10,837	-7.9	(-18.0, 3.4)	0.1	(-16.9, 20.6)	-11.2	(-22.2, 1.3)	0.53	-28.9	(-56.7, 16.9)	-6.7	(-17, 4.9)	0.31
	PM_{10}	9,914	6.3	(-1.9, 15.2)	10.4	(-3.7, 26.5)	4.6	(-4.9, 14.9)	0.94	-20.8	(-45.3, 14.7)	7.7	(-0.7, 16.9)	0.50
	$PM_{2.5}$	2,248	21.0	(3.9, 40.8)	24.8	(-2.4, 59.6)	18.9	(-0.3, 41.9)	0.62	-50.2	(-77.2, 8.9)	24.6	(6.8, 45.3)	0.15
	SO_2	10,301	1.5	(-8.4, 12.6)	0.2	(-16.9, 20.8)	1.7	(-9.9, 14.9)	0.28	0.4	(-37.2, 60.5)	1.4	(-8.8, 12.7)	0.98
Atrial Fibrillation	CO	9,660	-0.7	(-11.3, 11.1)	3.9	(-14.5, 26.2)	-2.9	(-15, 10.8)	0.27	-62.4	(-78.5, -34)	3.1	(-8.1, 15.6)	<0.01
	NO_2	10,526	-1.3	(-13.5, 12.5)	6.4	(-15.1, 33.3)	-4.5	(-18.1, 11.3)	0.51	-67.3	(-82.6, -38.6)	2.8	(-10, 17.5)	<0.01
	O_3	10,731	-7.9	(-18.0, 3.5)	1.3	(-16, 22.2)	-11.6	(-22.6, 0.9)	0.50	-33.5	(-60.1, 10.9)	-6.6	(-17, 5.1)	0.35
	PM_{10}	9,821	6.5	(-1.8, 15.4)	10.1	(-4.1, 26.4)	4.9	(-4.6, 15.3)	0.92	-28.1	(-51.2, 5.7)	8.2	(-0.3, 17.5)	0.27
	$PM_{2.5}$	2,230	21.0	(3.9, 41.0)	25.3	(-2.2, 60.5)	18.8	(-0.5, 41.8)	0.55	-49.1	(-77.1, 12.9)	24.5	(6.7, 45.3)	0.20
	SO_2	10,198	1.0	(-9.0, 12.1)	-1.4	(-18.4, 19.2)	10.1	(-2.5, 24.4)	0.28	-7.0	(-43.1, 51.9)	1.1	(-9.1, 12.4)	0.99
Pulmonary embolism	CO	10,404	-3.4	(-13.3, 7.6)	7.6	(-8.8, 26.9)	-10	(-21.4, 3.2)	0.48	-17.0	(-33.6, 3.9)	1.0	(-10.4, 13.9)	0.39
	NO_2	11,385	4.0	(-8.5, 18.2)	8.6	(-10.8, 32.1)	1.3	(-13.6, 18.6)	0.96	-5.4	(-27, 22.6)	6.8	(-7.4, 23.1)	0.26
	O_3	11,577	-7.0	(-16.8, 4.1)	-14.6	(-27.4, 0.6)	-1.8	(-14.2, 12.3)	0.31	-6.6	(-24.2, 15.1)	-7.3	(-18.1, 4.8)	0.62
	PM_{10}	10,372	0.5	(-7.2, 8.9)	-1.1	(-12.6, 11.9)	1.4	(-8.1, 12)	0.52	-2.7	(-17.1, 14.3)	1.5	(-7.1, 10.9)	0.92
	$PM_{2.5}$	2,401	20.2	(3.5, 39.7)	15.3	(-8.1, 44.6)	23.3	(2.9, 47.8)	0.98	7.3	(-20.3, 44.3)	23.8	(5, 45.8)	0.74
	SO_2	10,948	-0.3	(-9.9, 10.4)	-4.2	(-18.1, 12)	2.2	(-10.2, 16.3)	0.49	-5.9	(-23.7, 16)	1.1	(-9.8, 13.3)	0.33
Heart failure	CO	31,105	2.4	(-3.6, 8.9)	4.1	(-5.3, 14.4)	1.4	(-6.1, 9.3)	0.25	-7.0	(-27.4, 19)	2.9	(-3.3, 9.6)	0.62
	NO_2	33,733	-2.6	(-9.3, 4.6)	-2.6	(-12.8, 8.8)	-2.6	(-10.7, 6.3)	0.23	-4.0	(-27, 26.3)	-2.4	(-9.2, 5)	0.90
	O_3	34,404	3.4	(-3.1, 10.3)	-1.3	(-10.3, 8.6)	6.3	(-1.6, 14.8)	0.03	11.4	(-12.2, 41.2)	3.0	(-3.6, 10)	0.53
	PM_{10}	31,412	0	(-4.4, 4.6)	0.3	(-6.5, 7.5)	-0.2	(-5.6, 5.5)	0.90	-7.7	(-22.6, 10.2)	0.5	(-4, 5.3)	0.23
	$PM_{2.5}$	8,871	-3.2	(-10.5, 4.6)	-0.6	(-11.4, 11.7)	-5.0	(-13.6, 4.5)	0.55	10.2	(-16.1, 44.6)	-3.9	(-11.2, 4.1)	0.54
	SO_2	32,792	-3.4	(-8.8, 2.2)	-4.0	(-12.4, 5.1)	-3.1	(-9.8, 4)	0.34	-13.9	(-31.9, 8.9)	-2.6	(-8.1, 3.3)	0.22

IHD, ischaemic heart disease; MI, myocardial infarction; ONS, office of national statistics.
P value by 2-sided Wald test for overall interaction terms
Results of atrio-venticular conduction disorders are not shown due to the limited number of events.
[a] including MI and chronic IHD
[b] excluding conduction disorders and bradycardias, including atrial fibrillation

Table S4 Within-stratum correlation between pollutants: [a] HES analysis and [b] mortality analysis

[a] HES analysis

	CO	NO_2	O_3	PM_{10}	$PM_{2.5}$	SO_2
CO	1.0000	-	-	-	-	-
NO_2	0.6450	1.0000	-	-	-	-
O_3	-0.2973	-0.3489	1.0000	-	-	-
PM_{10}	0.4802	0.5445	0.0302	1.0000	-	-
$PM_{2.5}$	0.4752	0.5299	-0.0960	0.8611	1.0000	-
SO_2	0.3035	0.4554	-0.0849	0.4356	0.4078	1.0000

[b] mortality analysis

	CO	NO_2	O_3	PM_{10}	$PM_{2.5}$	SO_2
CO	1.0000	-	-	-	-	-
NO_2	0.6580	1.0000	-	-	-	-
O_3	-0.3024	-0.3290	1.0000	-	-	-
PM_{10}	0.4791	0.5474	0.0146	1.0000	-	-
$PM_{2.5}$	0.4754	0.5416	-0.0450	0.8715	1.0000	-
SO_2	0.2910	0.4633	-0.0853	0.4432	0.4333	1.0000

Figure S1. AURN monitoring sites for air pollution (red points, except roadside and curbside) and BADC sites for temperature (blue points) in 2003-2009. Gray dots show MINAP patient's residential addresses.

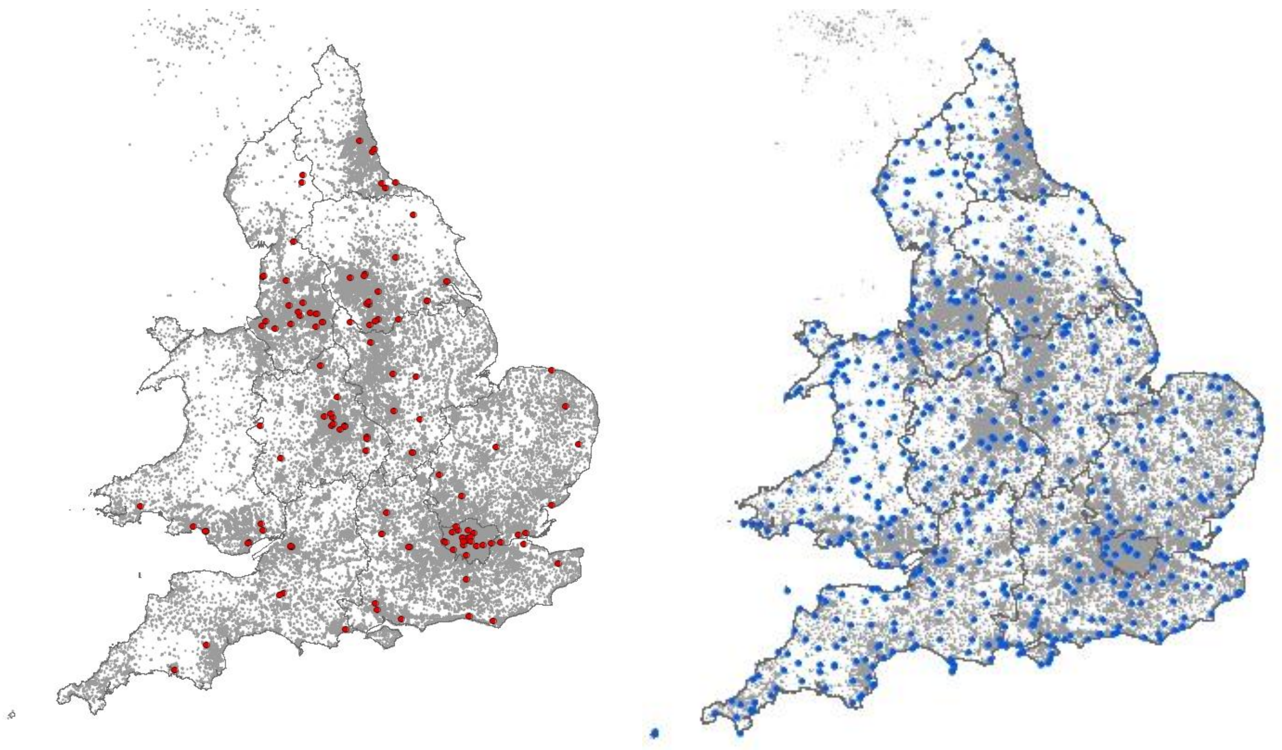

Figure S2. Correlations and mean differences of daily pollution and temperature values by distance. Points represent agreement between all possible combinations of 2 monitors. Figures show agreement separately by season (April-September and October-March) and by distances (within 500km and then just within 50km). $PM_{2.5}$ is not shown due to small number of monitoring stations.

CO (daily mean, mg/m^3): 35 sites

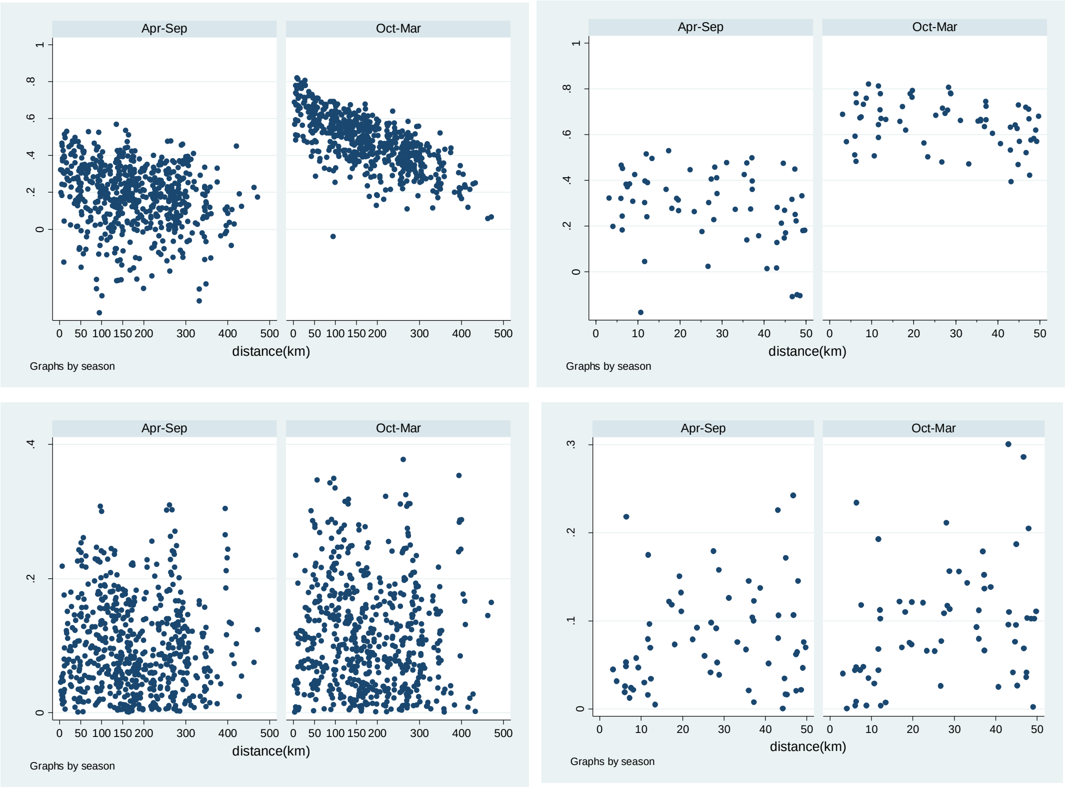

NO_2 (daily mean, mg/m^3): 50 sites

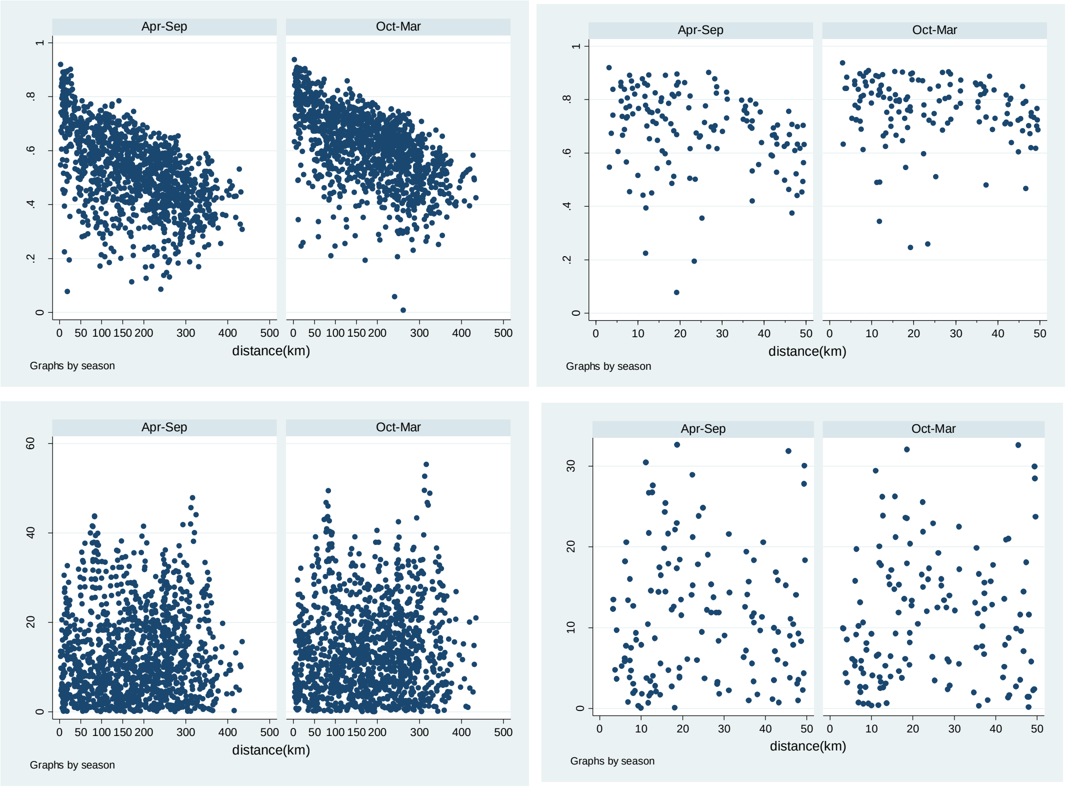

O3 (daily mean, mg/m3): 60 sites

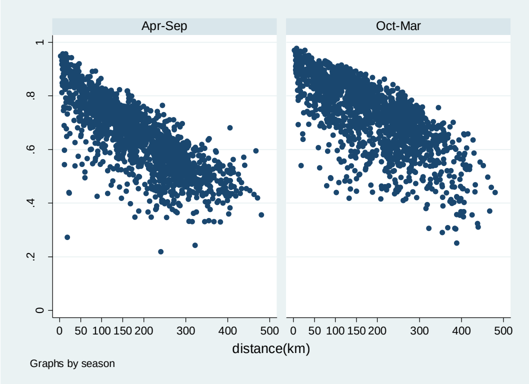

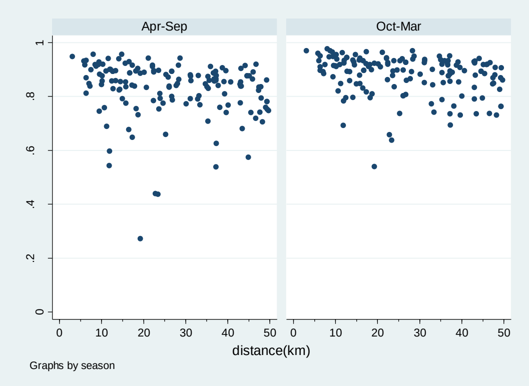

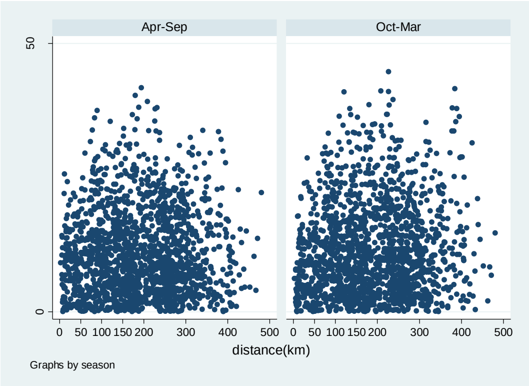

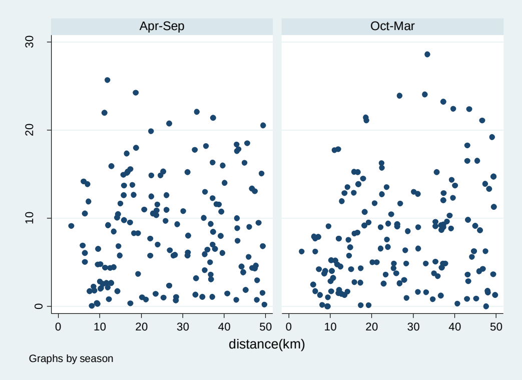

PM_{10} (daily mean, $\mu g/m^3$): 36 sites

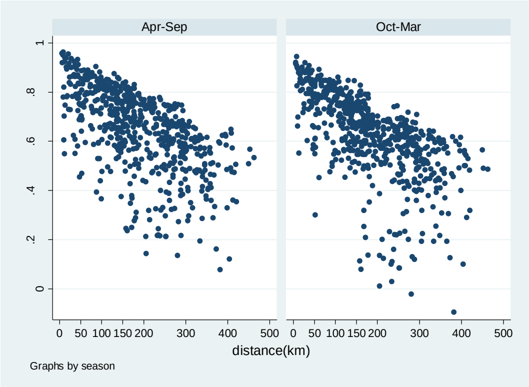

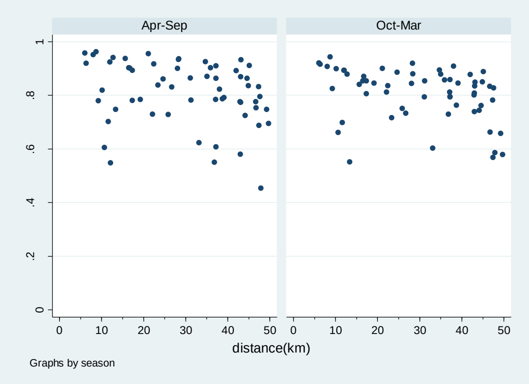

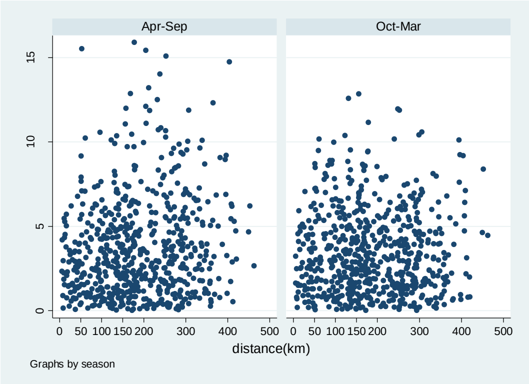

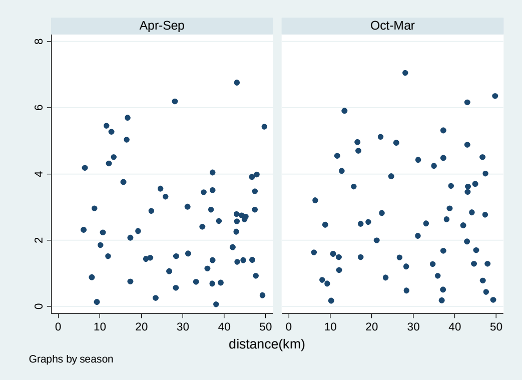

SO_2 (daily mean, mg/m^3): 45 sites

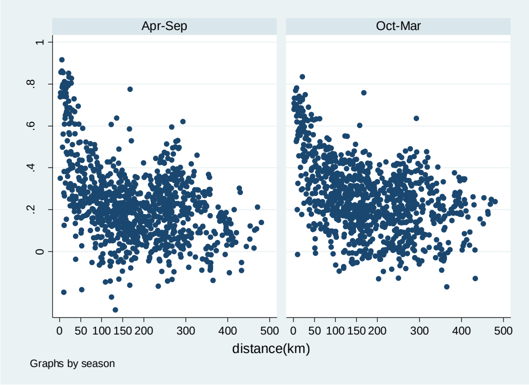

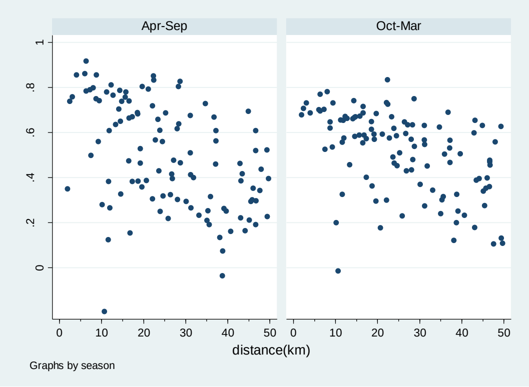

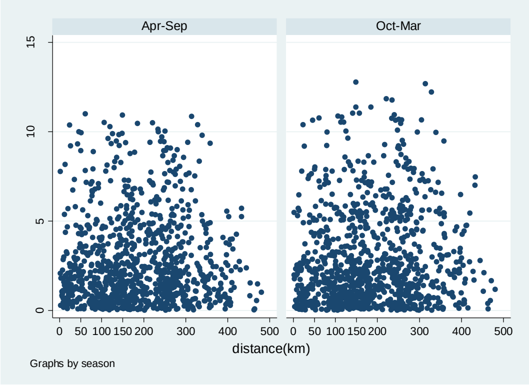

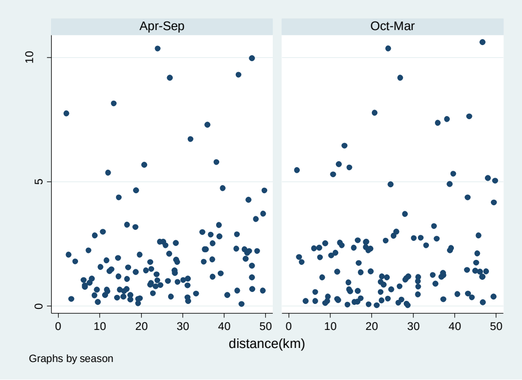

Temperature (daily mean, degrees C): 254 sites

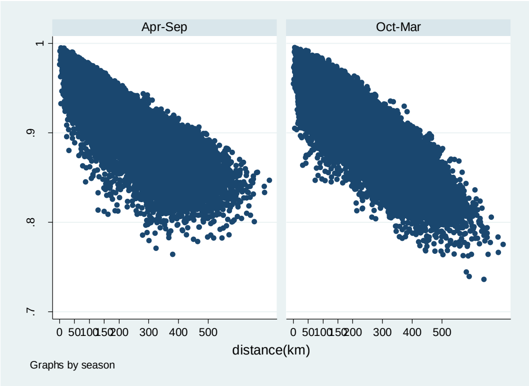

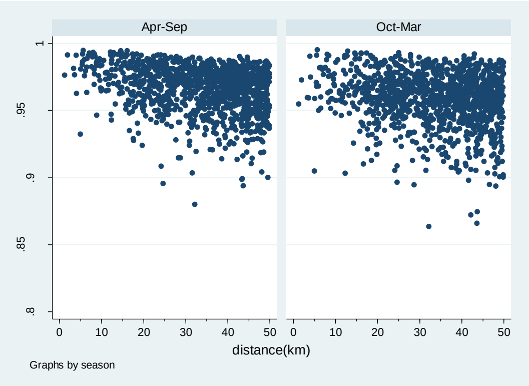

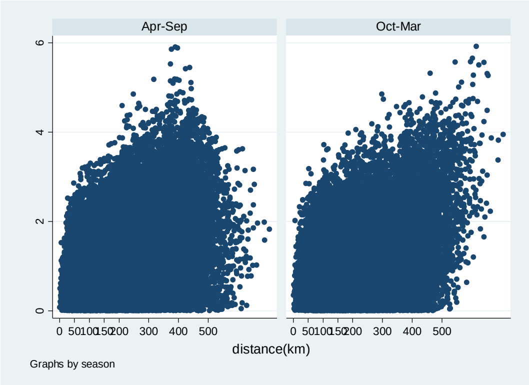

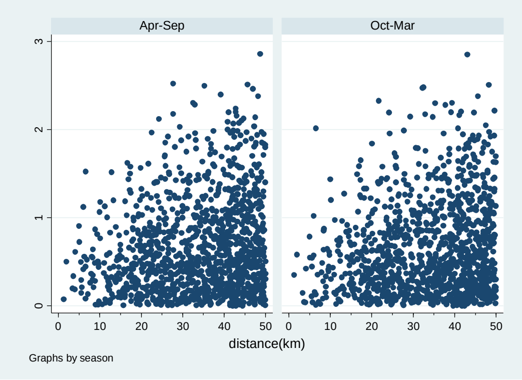

Figure S3. Effects of NO_2 at lags 0-4days on [a] STEMI and [b] non-STEMI diagnosis by risk factors. Column on extreme right shows *P* value from interactions model.

[a]

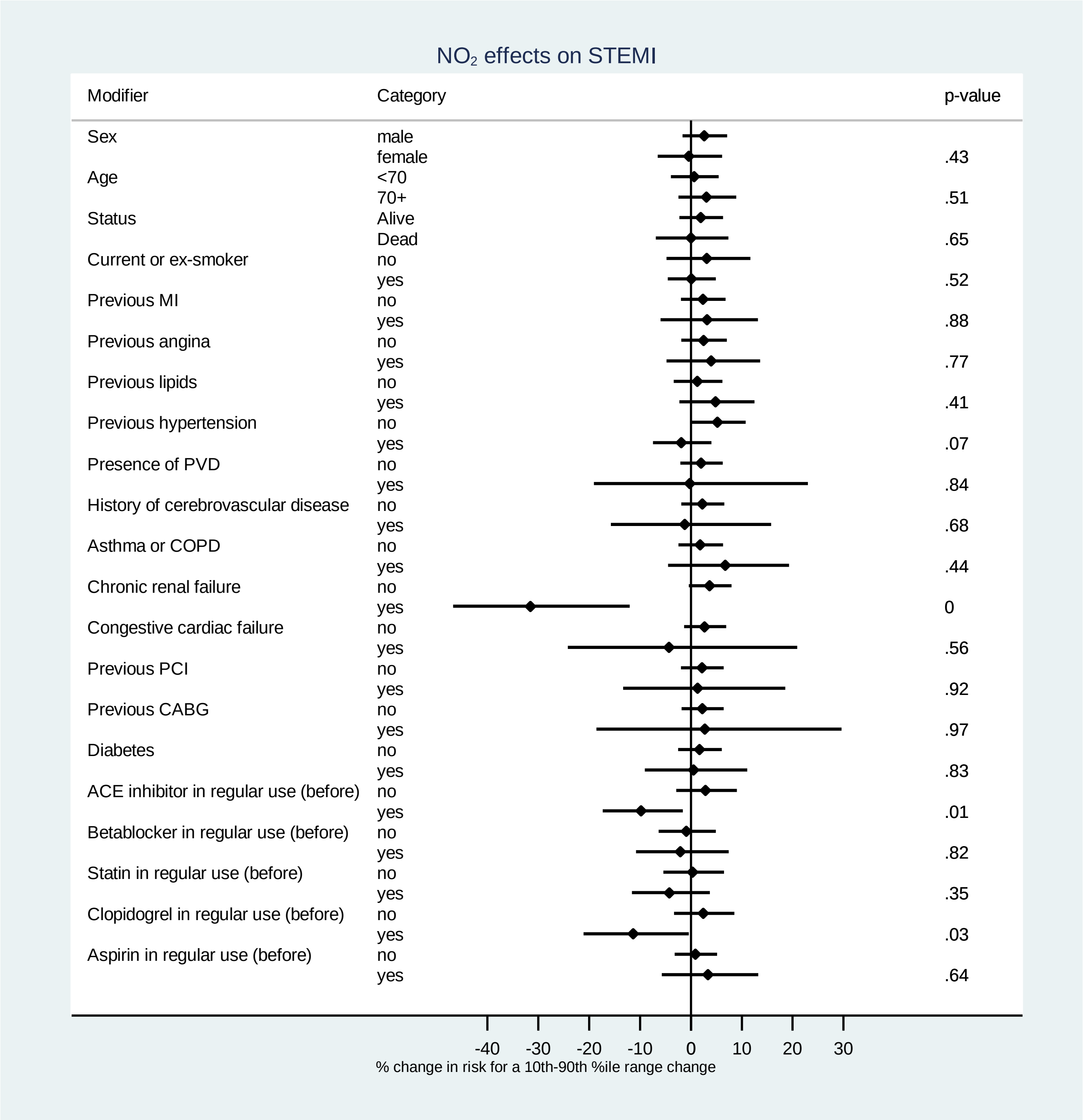

ACE, angiotensio converting enzyme; PVD, peripheral vascular disease; PCI, percutaneous coronary intervention; CABG, coronary artery bypass graft;

[b]

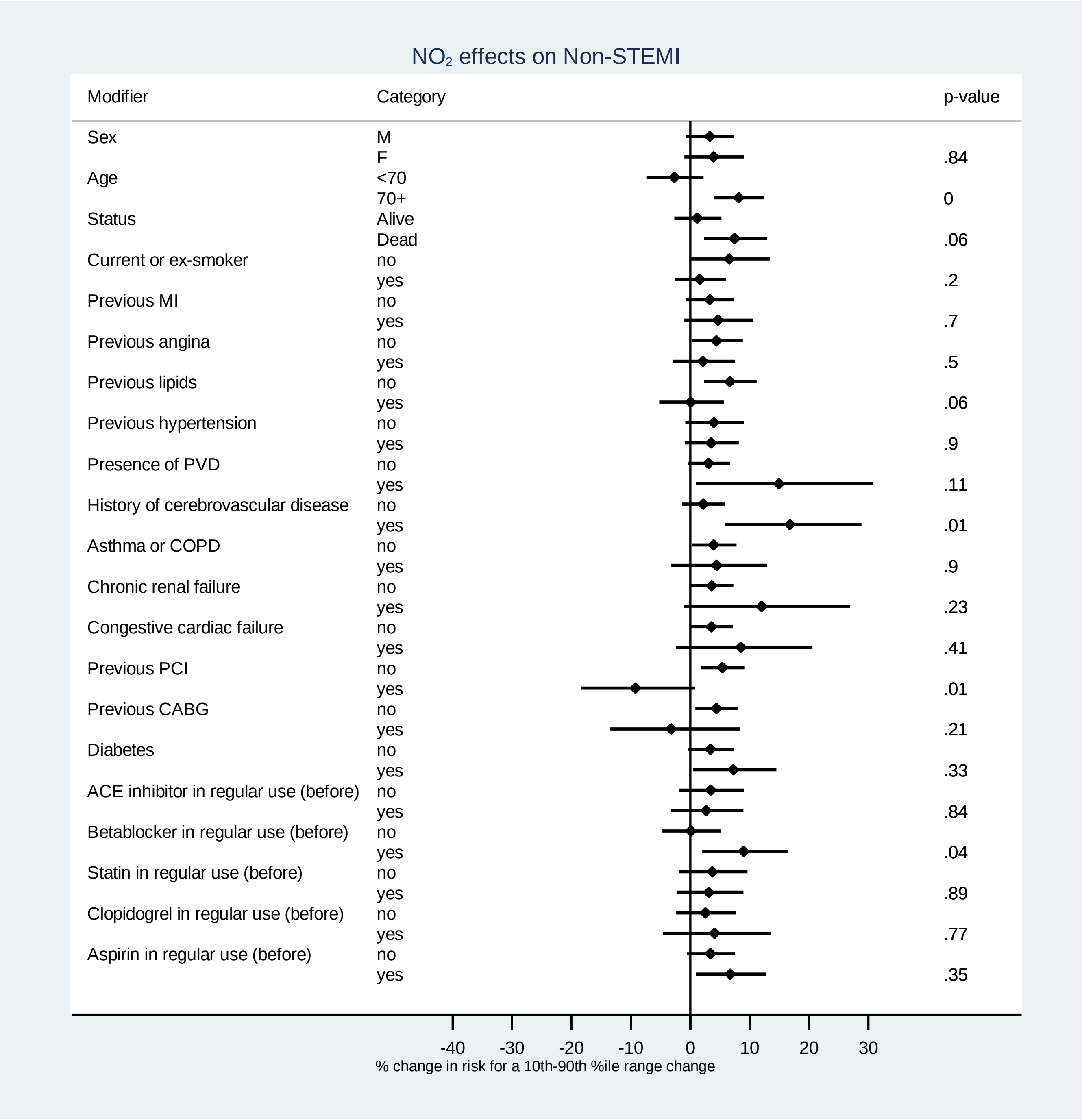

ACE, angiotensio converting enzyme; PVD, peripheral vascular disease; PCI, percutaneous coronary intervention; CABG, coronary artery bypass graft;

Figure S4. Effects of SO_2 at lags 0-4days on [a] STEMI and [b] non-STEMI diagnosis by risk factors. Column on extreme right shows *P* value from interactions model.

[a]

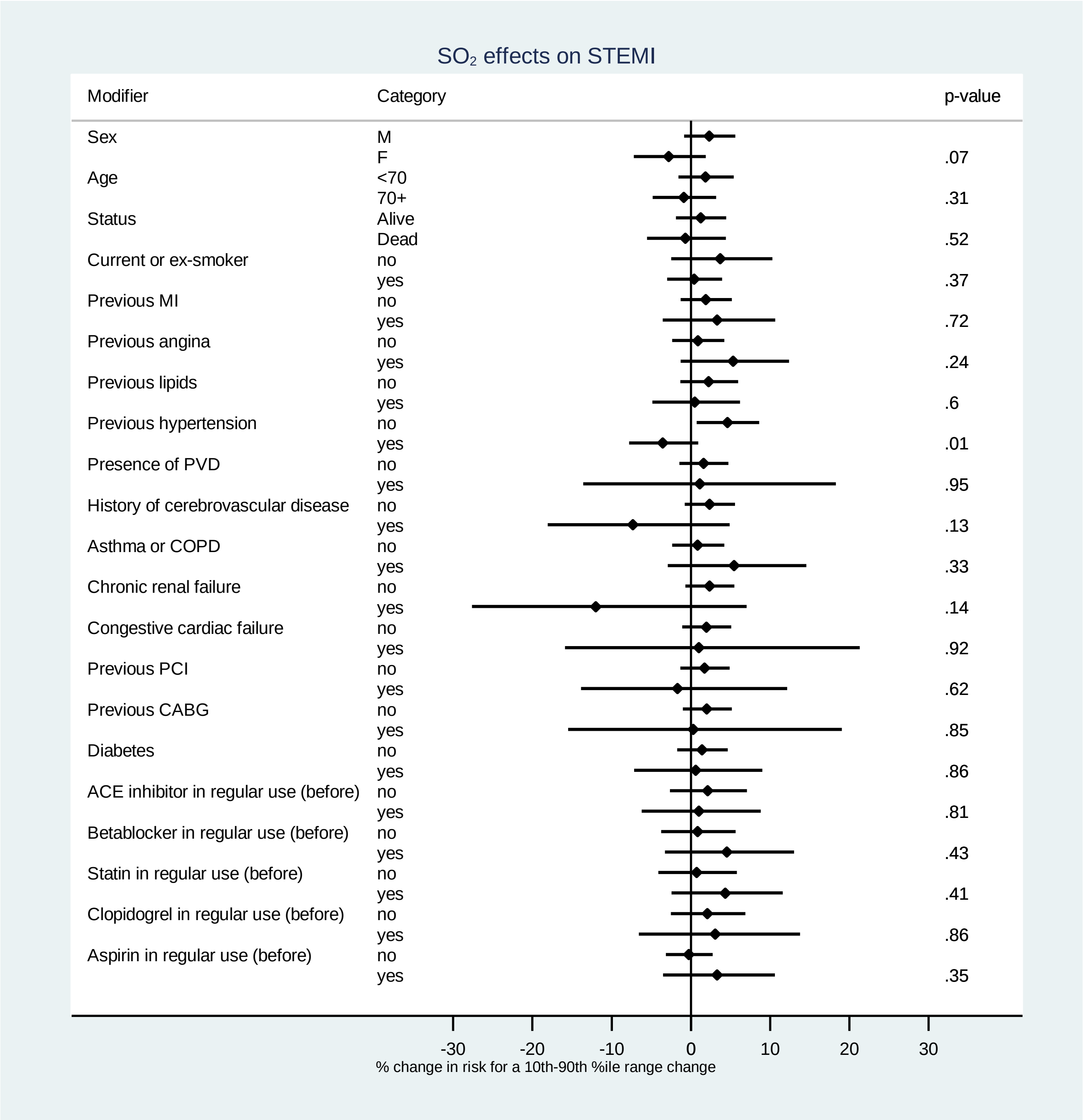

ACE, angiotensio converting enzyme; PVD, peripheral vascular disease; PCI, percutaneous coronary intervention; CABG, coronary artery bypass graft;

[b]

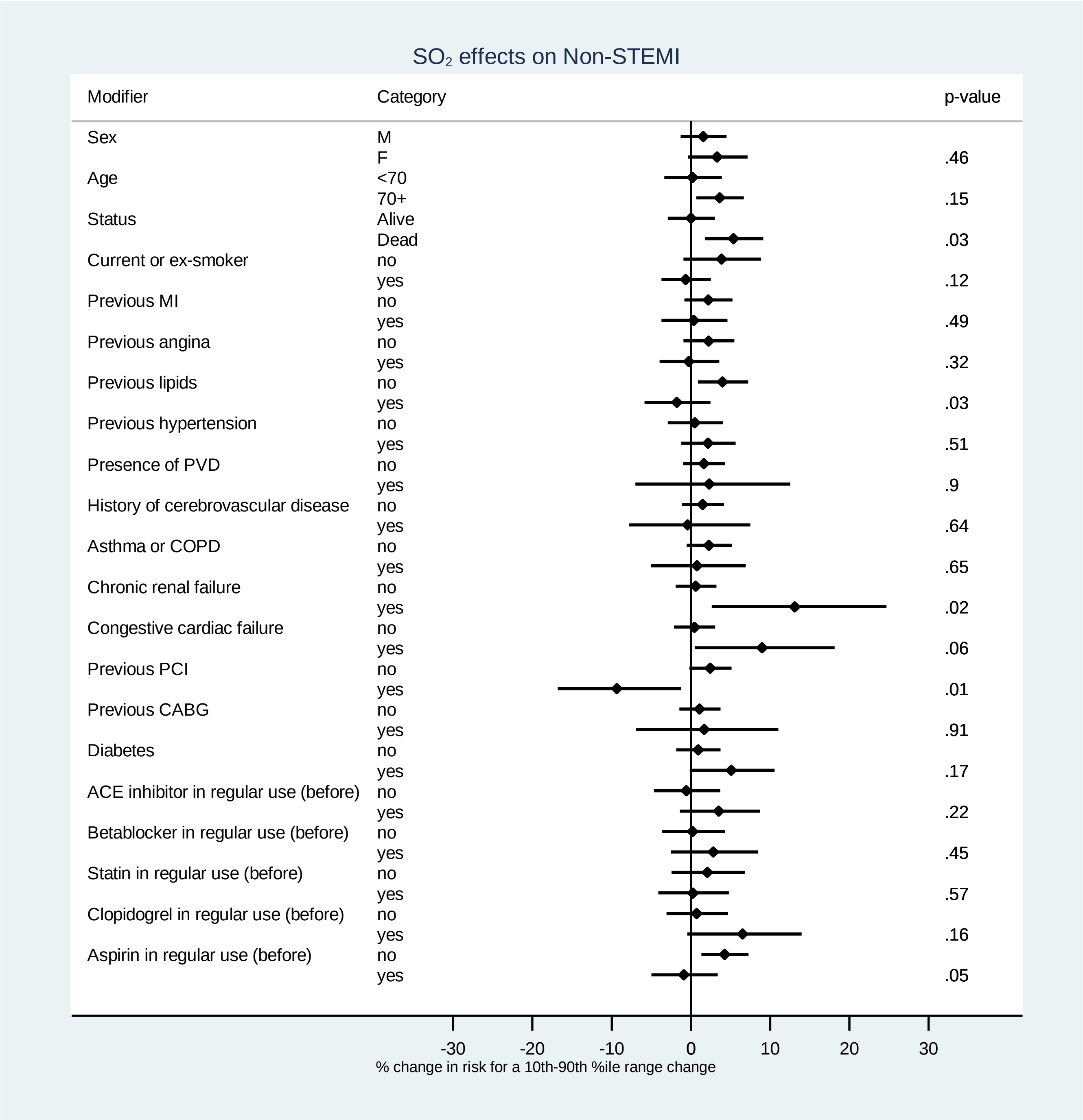

ACE, angiotensio converting enzyme; PVD, peripheral vascular disease; PCI, percutaneous coronary intervention; CABG, coronary artery bypass graft;

Figure S5. Percentage change (95%CI) in risk of cardiovascular events for a 10th-90th percentile range change in pollutant at lags 0-1 days and lags 0-4 days. 10th-90th percentile ranges in pollutant vary in databases: [a] MINAP 2003-2009, [b] HES 2003-2008, and [c] ONS mortality 2003-2006. AVCD, MI, and IHD represent Atrio-venticular conduction disorder, myocardial infarction, and ischaemic heart disease respectively.

[a]

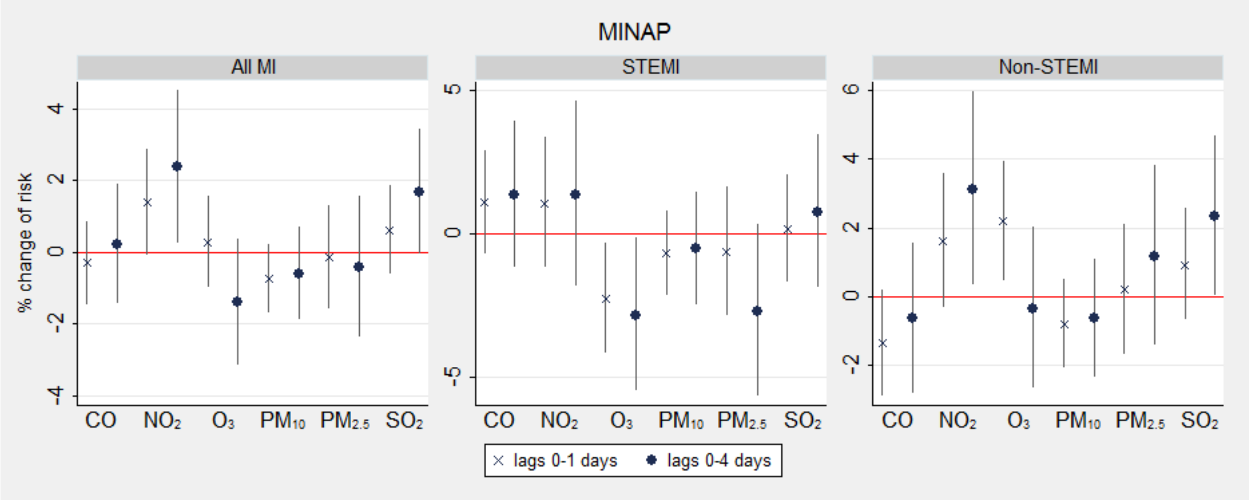

[b]

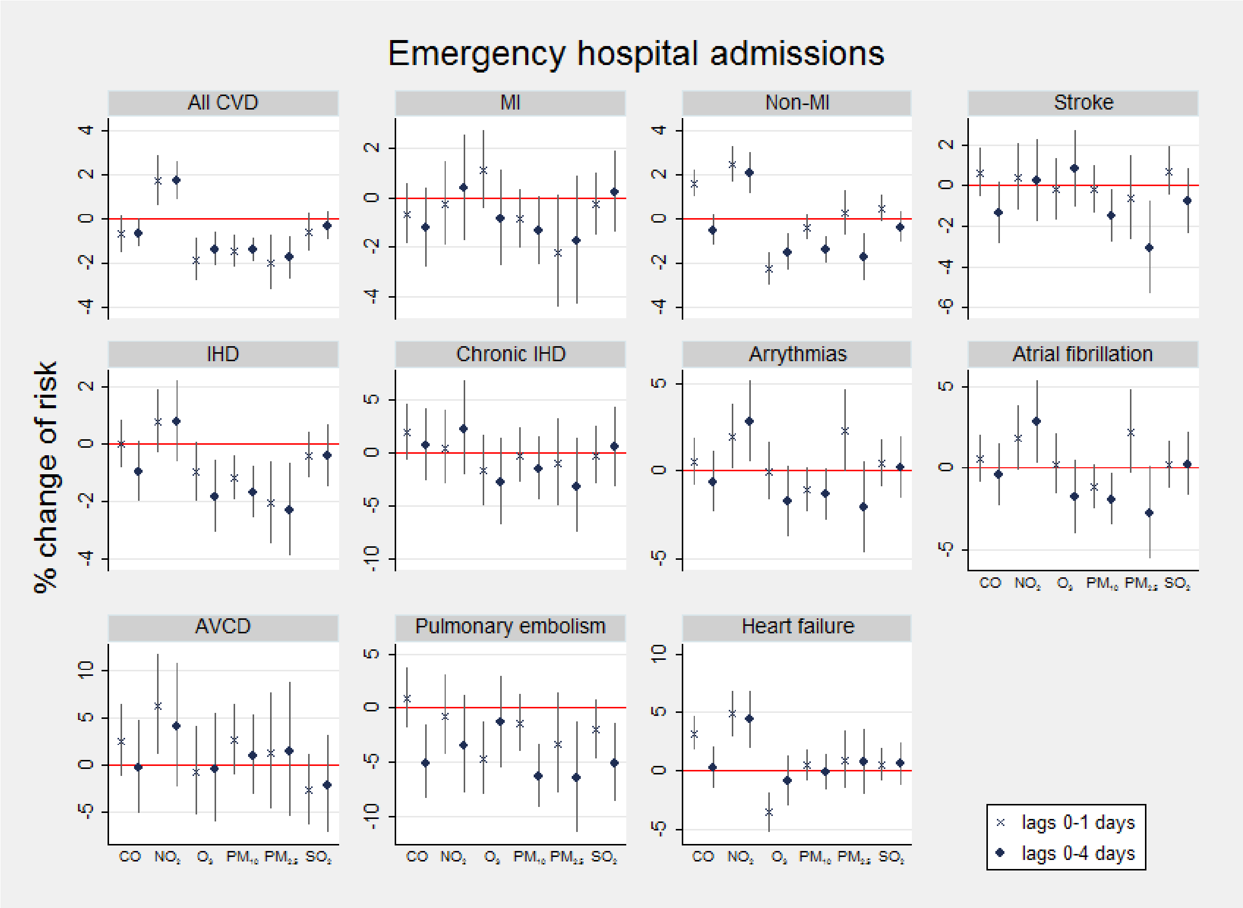

[c]

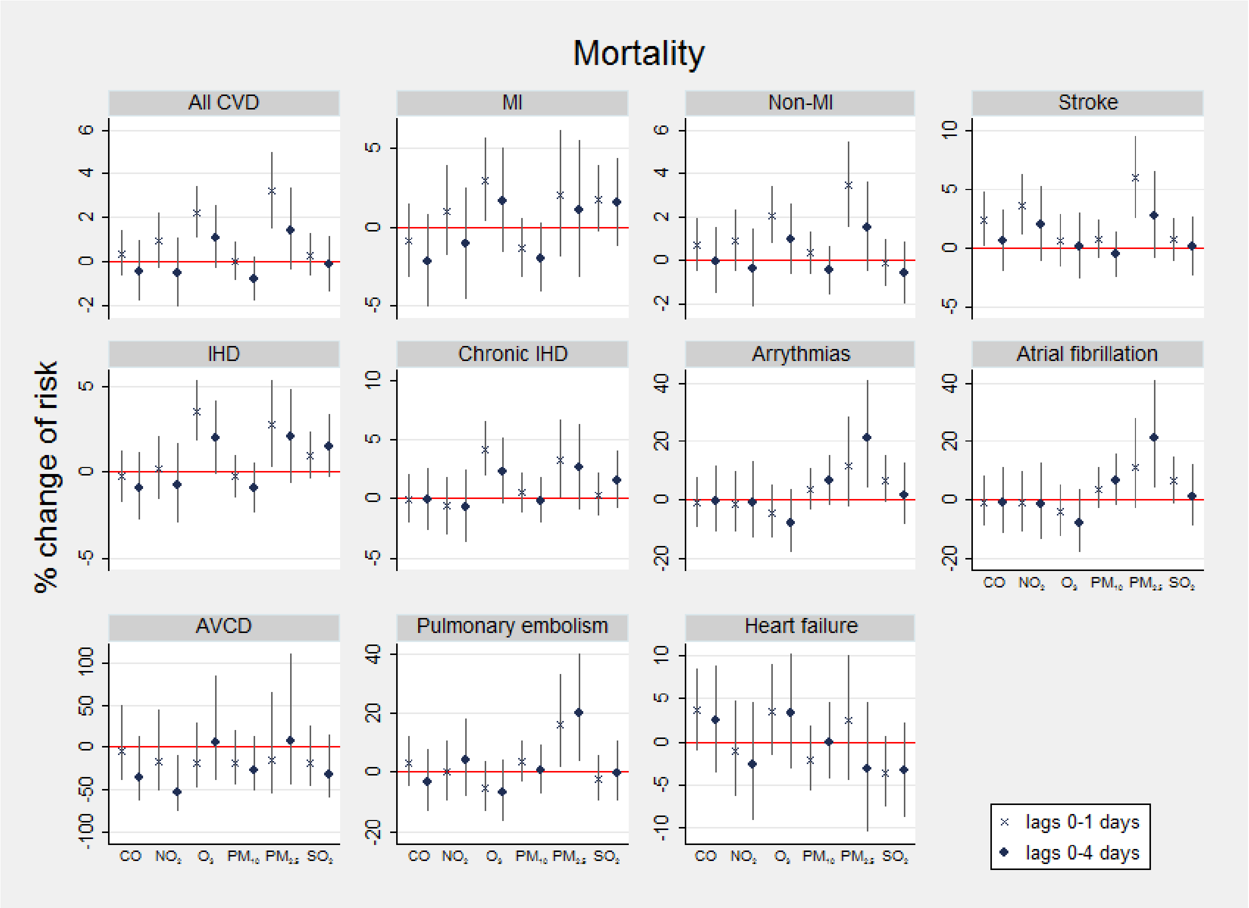
Mortality
All CVD
MI
Non-MI
Stroke
IHD
Chronic IHD
Arrythmias
Atrial fibrillation
AVCD
Pulmonary embolism
Heart failure
% change of risk
CO NO$_2$ O$_3$ PM$_{10}$ PM$_{2.5}$ SO$_2$
lags 0-1 days
lags 0-4 days
